# Rab27a Contributes to Cathepsin S Secretion in Lacrimal Gland Acinar Cells

**DOI:** 10.3390/ijms22041630

**Published:** 2021-02-05

**Authors:** Runzhong Fu, Maria C. Edman, Sarah F. Hamm-Alvarez

**Affiliations:** 1 Department of Pharmacology and Pharmaceutical Sciences, School of Pharmacy, University of Southern California, Los Angeles, CA 90033, USA; runzhonf@usc.edu; 2 Department of Ophthalmology, Roski Eye Institute, Keck School of Medicine, University of Southern California, Los Angeles, CA 90033, USA; edman@usc.edu

**Keywords:** Sjögren’s syndrome, Cathepsin S, Rab27a, lacrimal gland, rab proteins, acinar cells, regulated secretion, endolysosomal secretion

## Abstract

Altered lacrimal gland (LG) secretion is a feature of autoimmune dacryoadenitis in Sjögren’s syndrome (SS). Cathepsin S (CTSS) is increased in tears of SS patients, which may contribute to disease. Rab3D and Rab27a/b isoforms are effectors of exocytosis in LG, but Rab27a is poorly studied. To investigate whether Rab27a mediates CTSS secretion, we utilized quantitative confocal fluorescence microscopy of LG from SS-model male NOD and control male BALB/c mice, showing that Rab27a-enriched vesicles containing CTSS were increased in NOD mouse LG. Live-cell imaging of cultured lacrimal gland acinar cells (LGAC) transduced with adenovirus encoding wild-type (WT) mCFP-Rab27a revealed carbachol-stimulated fusion and depletion of mCFP-Rab27a-enriched vesicles. LGAC transduced with dominant-negative (DN) mCFP-Rab27a exhibited significantly reduced carbachol-stimulated CTSS secretion by 0.5-fold and β-hexosaminidase by 0.3-fold, relative to stimulated LGAC transduced with WT mCFP-Rab27a. Colocalization of Rab27a and endolysosomal markers (Rab7, Lamp2) with the apical membrane was increased in both stimulated BALB/c and NOD mouse LG, but the extent of colocalization was much greater in NOD mouse LG. Following stimulation, Rab27a colocalization with endolysosomal membranes was decreased. In conclusion, Rab27a participates in CTSS secretion in LGAC though the major regulated pathway, and through a novel endolysosomal pathway that is increased in SS.

## 1. Introduction

The lacrimal gland (LG) is an exocrine gland that secretes lacrimal fluid, essential for maintaining ocular surface health [1]. Lacrimal fluid is a complex mixture of water, electrolytes, hydrolases, and proteins including antibodies, growth factors, cytokines, proteases and protease inhibitors [1]. The LG is primarily composed of lacrimal gland acinar cells (LGAC), ductal cells, myoepithelial cells, and immune cells. Constituting >80% of the glandular mass, LGAC play a principal role in lacrimal fluid secretion. LGAC are polarized secretory epithelial cells, with distinct apical and basolateral membranes connected by tight junctions close to the apical membrane region, which form tubuloacinar structures. The basolateral membrane contains many ion channels, ion transport proteins, and receptors for neurotransmitters, neuropeptides, and growth factors [1,2]. Activation of basolateral receptors triggers intracellular signaling, resulting in mobilization of trafficking of stored secretory vesicles to the apical plasma membrane (APM), where they fuse and release their contents [2].

Lacrimal gland dysfunction is one of the main causes of aqueous-deficient dry eye disease (ADDE). A major subtype of severe ADDE is associated with Sjögren’s syndrome (SS) [3]. SS is a systemic autoimmune disease associated with lymphocytic infiltration of exocrine glands [4], and primarily affecting LG and salivary glands (SG). The ensuing reduced tear and saliva production, respectively, are classic symptoms of SS, accompanied by associated complications such as blurred vision, corneal damage, dental caries, loss of taste sensation and oral thrush [4]. Besides exocrine morbidity, SS patients may experience other general and systemic manifestations including weight loss, fatigue, inflammation of internal organs and B-cell lymphoma [4,5]. Inflammation of the LG not only affects tear flow, but also alters tear composition, producing more inflammatory and proteolytic tears [6,7,8,9]. The altered tears may further damage the ocular surface by evoking apoptosis and autophagy in ocular surface epithelial cells [10]. During inflammation, pro-inflammatory cytokines released into the tear film also disturb afferent and efferent sensory nerves, further compromising tear secretion [2,7,11]. Despite impacting 0.5–1% of the general population, there are currently no specific therapies for SS [12]. Current treatments for SS-associated ADDE focus on alleviation of ocular symptoms in combination with systemic immunomodulatory therapies [13]. Therefore, it is of clinical relevance to understand the mechanisms in the LG that trigger ocular inflammation and evoke secretion of inflammatory and proteolytic tears.

Cathepsin S (CTSS) is a lysosomal cysteine protease associated with many physiological processes, including extracellular matrix degradation [14], major histocompatibility complex II (MHC II) processing in antigen presentation [15] and protein catabolism [15,16,17,18]. Dysregulation of CTSS is implicated in multiple autoimmune diseases [14], including rheumatoid arthritis [19], systemic lupus erythematosus [20], multiple sclerosis [21] and SS [19,20,22,23]. CTSS activity is significantly elevated in tears of SS patients compared to tears from patients with non-SS dry eye or other autoimmune diseases [23]. CTSS activity is also significantly increased in the LG and tears of an SS murine model, the male NOD mouse [17,24,25]. Male NOD mice share many characteristics of ocular manifestation with SS patients including reduced tear flow [17,23,24,25,26], lymphocytic infiltration of the LG [17,24,25], development of a proteolytic and inflammatory tear film [17,27], loss of extracellular matrix [24], and altered acinar Rab3D distribution [26,28]. 

Elevated CTSS in tears and LG may disrupt surface homeostasis and reinforce lymphocyte recruitment [18]. In vitro studies have shown that CTSS, at concentrations equal to those found in SS patient tears, induces release of pro-inflammatory cytokines from cultured corneal epithelial cells [29]. CTSS is also involved in activation and release of the chemokine, CX3CL1, from LG acinar cells (LGAC) and into tears in male NOD mice [17]. In vivo inhibition of CTSS activity in the male NOD mouse significantly reduced lymphatic infiltration of the LG and increased tear flow [29]. Hence, elevated CTSS may be involved in the etiology of SS. Despite multiple studies substantiating the pathological functions of CTSS in SS, the mechanisms responsible for the altered intracellular trafficking of CTSS in LGAC in SS remain unclear. 

Regulated exocytosis is well-studied as the principal secretory mechanism in LGAC; newly-synthesized proteins, packaged in large secretory vesicles (SV), are mobilized for exocytosis at the APM through activation of various signaling pathways. Proteins secreted through regulated exocytosis include lactoferrin, β-hexosaminidase (β-hex) and peroxidase. Rab proteins are critical regulators of regulated exocytosis. As part of the Ras superfamily of small GTPases, Rab proteins can modulate membrane association and disassociation through regulated binding and hydrolysis of GTP [30,31]. The Rab3 and Rab27 subfamilies are commonly associated with SV exocytosis in neurons, endocrine, exocrine and immune cells [32]. Rab3D is the most highly expressed Rab3 protein in LGAC, and appears to modulate regulated exocytosis through regulation of SV maturation [33] and premature fusion [34]. Analysis of Rab3D distribution in LGAC by immunofluorescence reveals its enrichment on an abundant network of large (0.5–1 μm) apparent SV that are depleted following stimulation of secretion in healthy LGAC. The SS model male NOD mice exhibit reduced expression and altered LGAC distribution of Rab3D, concurrent with increased tear CTSS [26]. Previous work has shown that tear CTSS activity is reduced in mice lacking functional Rab27 isoforms and, moreover, is significantly increased in tears of Rab3D knockout mice which may exhibit a compensatory increased function of Rab27 isoforms [26]. These findings implicate Rab27 isoforms in CTSS secretion. 

Rab27 has two isoforms that share 71% homology in amino acid sequence: Rab27a and Rab27b [35]. Both isoforms are expressed on apparent SVs in pancreatic acini [36,37,38], parotid acini [39], and LGACs [26]. Rab27b is linked to regulated exocytosis in pancreatic [36] and parotid acinar cells [39], as well as in LGAC [26,40], since expression of dominant-negative (DN) Rab27b in LGAC significantly impairs the process of stimulated secretion. The role of Rab27a in regulated exocytosis has been extensively studied in various professional secretory cells including pancreatic and parotid acinar cells, melanocytes and cytotoxic T-lymphocytes [41]. Rab27a has been implicated in secretion from endosomes and lysosomes in certain cell types through direct fusion of multivesicular bodies (MVB) [42,43] and lysosomes with the plasma membrane [44,45]. Rab27a is also reported to modulate amylase release through a minor endolysosomal secretory pathway in mouse pancreatic acinar cells [37]. Despite findings showing partial colocalization of Rab27a on apical SVs enriched in Rab27b and Rab3D [26], its functional role during exocytosis in LGAC is yet unclear.

Given our previous functional findings implicating Rab27 isoforms in CTSS trafficking in LGAC [26], we explore here the hypothesis that Rab27a is a key regulator of CTSS secretion. To do this, we have demonstrated increased Rab27a-enriched vesicles enriched in CTSS in LGAC from the SS disease model, male NOD mouse LG, relative to control male BALB/c mouse LG. We developed WT and DN mCFP-Rab27a construct for transduction of primary cultured LGAC. Use of these constructs demonstrated carbachol (CCh)-induced stimulation of fusion and subsequent depletion of vesicles labeled with WT mCFP-Rab27a, as well as reduced CCh-stimulated exocytosis of CTSS and also β-hex in DN mCFP-Rab27a-transduced LGAC relative to LGAC transduced with WT mCFP-Rab27a. We have also shown a significant enrichment of endolysosomal membranes and Rab27a at the APM region of LGAC in stimulated male BALB/c and NOD mouse LG, which is more prominent for endolysosomes in NOD mouse LGAC. Rab27a colocalization with endolysosomal membranes was decreased with topical CCh stimulation, suggesting their fusion and release at the APM. We conclude by proposing that Rab27a is involved both in the major regulated secretory pathway, working in concert with Rab3D, as well as in a novel regulated endolysosomal secretory pathway, the activity of which is increased in SS-associated ADDE.

## 2. Results

### 2.1. CTSS and Rab27a Are Enriched in the Same Vesicles in LGAC from Male NOD Mice

**Figure 1:**
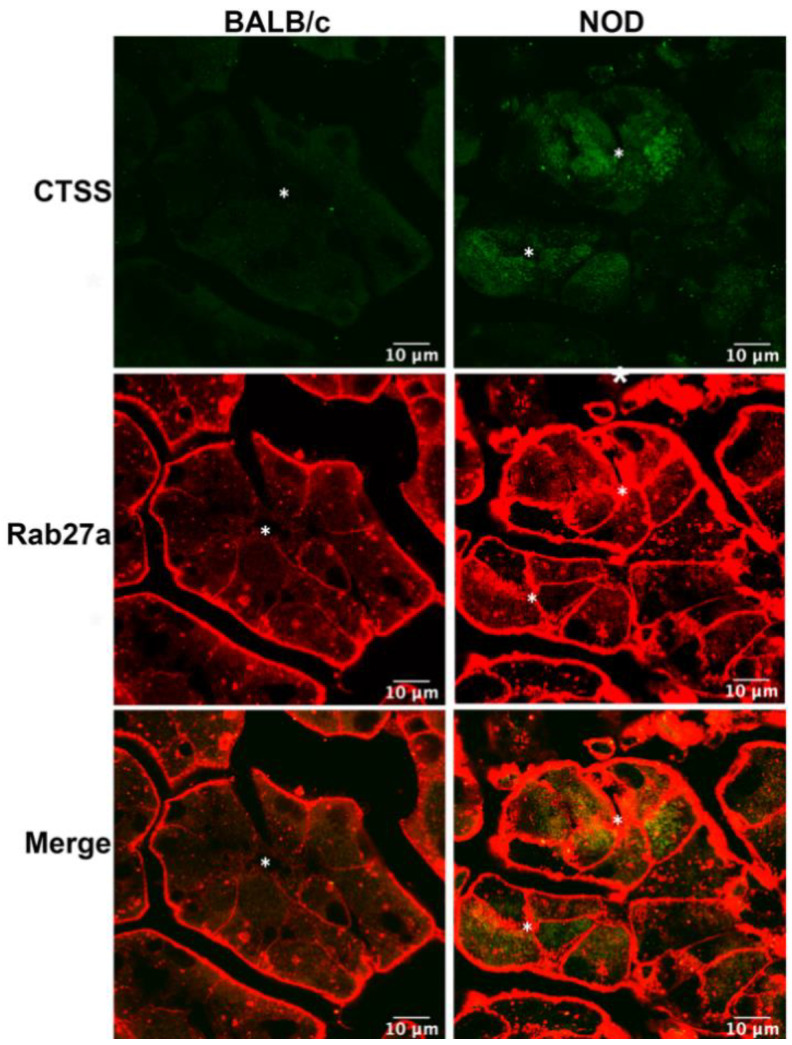
Increased enrichment of CTSS and Rab27a in vesicles in LGAC from male NOD mice. CTSS and Rab27a distribution in LGAC from LG from 16–17 week male BALB/c and NOD LG sections were characterized by immunofluorescence. CTSS immunofluorescence intensity (green) was clearly increased in NOD mouse LG sections, while also showing an increased colocalization with Rab27a (red) in the subapical area. Staining adjacent to the plasma membrane was observed for Rab27a due to secondary anti-mouse antibody reaction with residual interstitial immunoglobulins. In order to visualize intracellular Rab27a above this high interstitial background, the fluorescence signal in the interstitium was acquired under conditions of saturation. N = 4, N = number of mice. *, Lumen.

We have previously reported increased CTSS activity in the tears of male NOD mouse, as well as enhanced CTSS gene and protein expression in the LG with disease progression [24]. Analysis of primary cultured LGAC transduced with recombinant Ad-CTSS-GFP also showed that exogenous CTSS was first sorted into endolysosomes upon synthesis, and was eventually trafficked to subapically-enriched vesicles enriched in Rab27b [26]. 3D-structured illumination microscopy showed that Rab27a was partially colocalized with Rab27b on subapical SV [26]. In this study we utilized 16–17 week old male NOD mice as a model of SS-associated dacryoadenitis, which exhibits significant ocular manifestations of disease, without exhibiting hyperglycemia. To determine whether CTSS was detected in Rab27a-enriched subapical vesicles in diseased LGAC, we analyzed LG tissue from male BALB/c and NOD mice labeled with antibodies to CTSS and Rab27a for indirect immunofluorescence, as shown in Figure 1. In addition to its elevated expression in male NOD LG, as we have previously demonstrated [17,24,26], CTSS distribution is also altered. While showing the enrichment in small puncta detected throughout the cytoplasm seen in LGAC in BALB/c mouse LG sections, CTSS showed an increased abundance and striking accumulation beneath both apical and basolateral membranes in NOD mouse LG. Notably, the increased apically-enriched CTSS in the diseased male NOD mouse LGAC was colocalized with subapical Rab27a, suggesting that the increased CTSS stores accumulating in these cells were enriched in Rab27a-enriched vesicles. 

### 2.2. In Male NOD Mouse LG, Rab27a-Enriched Vesicles Are Subapically Increased and Their Size Decreased, Relative to Rab27a-Enriched Vesicles in Healthy Male BALB/c Mouse LG

**Figure 2:**
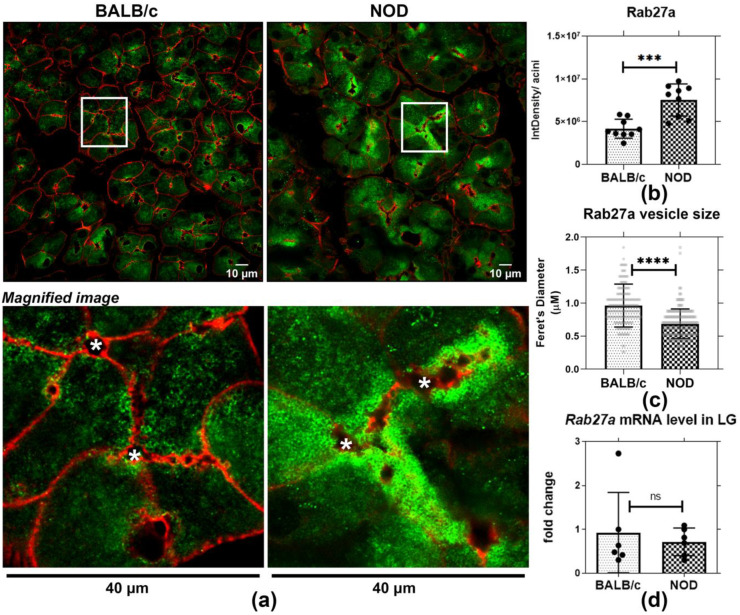
Increased accumulation on subapically-enriched vesicles and reduced vesicle diameter characterizes Rab27a-enriched vesicles in male NOD mouse. Tissue sections from 3–4 mice per strain were quantified, 3 ROI were obtained from each section. (**a**) Indirect immunofluorescence microscopy of Rab27a labeling (green) revealed increased accumulation of Rab27a-enriched vesicles near the apical lumen. (**b**) The increased intracellular accumulation of Rab27a was quantified per acinus, and was increased in male NOD mouse acini relative to BALB/c mouse acini. ***, *p* = 0.0003 (N = 4). Red, actin filaments. Integrated density of three acini were quantified and averaged in each ROI, presented as points on the graph. (**c**) Feret’s diameter of Rab27a-enriched vesicles was significantly decreased in LG sections from NOD mice relative to vesicles in LG sections from BALB/c mice. ****, *p* ≤ 0.0001 (N = 4). 30–40 vesicles from three acini were quantified from ROI, presented as points. (**d**) No significant changes in *Rab27a* gene expression were detected in NOD versus BALB/c mouse LG. ns, *p* = 0.6057 (N = 6). N = mice per group. *, Lumen.

To further evaluate Rab27a distribution and expression in healthy and diseased LGAC, we quantified the intensity of immunostaining/acinus as well as the size of Rab27a-enriched vesicles in LGAC from male BALB/c and NOD mouse LG sections. We also analyzed Rab27a expression in the same samples through qRT-PCR. Confocal microscopy imaging of indirect immunofluorescence demonstrated that Rab27a-enriched vesicles were present in both male BALB/c and NOD mouse LGAC near apical and basolateral membranes (Figure 2a). Quantification of Rab27a immunofluorescence per acinar cell using the corrected total cell fluorescence (CTCF) method [46,47] revealed that Rab27 accumulation was significantly elevated in NOD mouse LGAC, as shown in Figure 2b. Moreover, the increase was apparent, by visual inspection to be more prominent in the subapical area. No change was observed in *Rab27a* gene expression through qPCR (Figure 2d). Since Rab27a undergoes a GTP-GDP cycle that controls its membrane association and dissociation [48], the increased Rab27a intensity associated with membranes may be related to protein activation and increased membrane recruitment rather than altered expression. Quantification of vesicle size also showed a significant reduction in Rab27a-enriched vesicle diameter in sections from male NOD LG relative to BALB/c mouse LG (Figure 2c). This decreased vesicle size may result from the inability of Rab27a-enriched vesicles to sustain normal trafficking and possibly, merger with subapical Rab3D-enriched SV that are decreased in diseased male NOD mouse LGAC [26]. It has been reported that Rab3D is required to maintain normally-sized SV [34]. In other secretory cells, a reduction in SV size is associated with reorganization of contents and/or vesicle condensation [49]. These observations suggested possible alteration in vesicle content as well a function of Rab27a-enriched apical vesicles in NOD mouse.

### 2.3. Ad-mCFP-Rab27a Construct Design and Characterization

**Figure 3:**
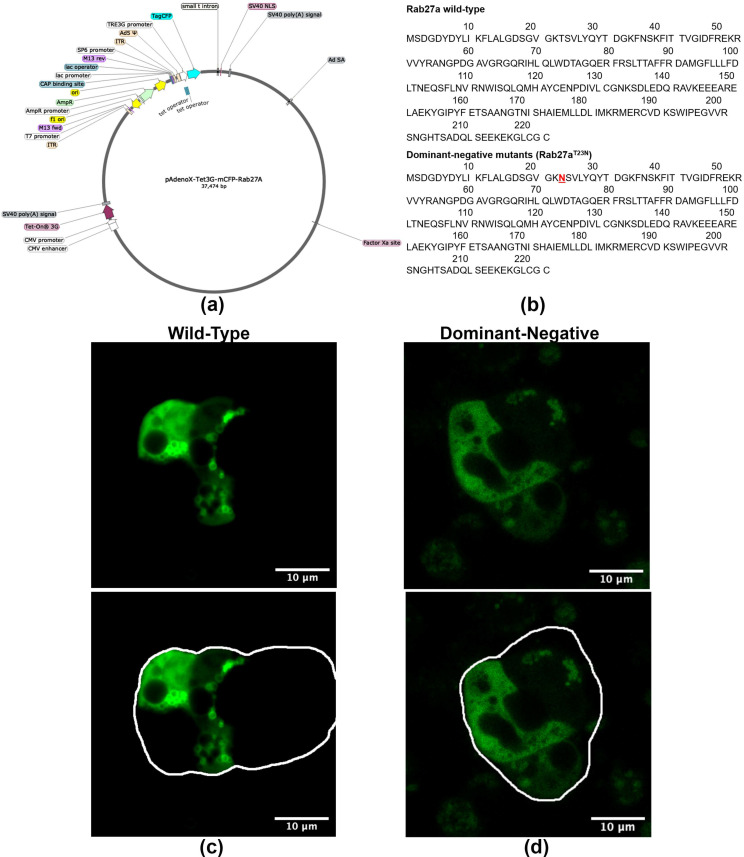
WT and DN Ad-mCFP-Rab27a constructs and characterization. (**a**) Tet-on WT and DN Ad-mCFP-Rab27a vectors were generated by subcloning the mCFP-Rab27a inserts into the in-fusion cloning site of linearized pAdenoX-Tet-3G vector and packaged in 293a cells. (**b**) Amino acid sequences of WT Rab27a and DN Rab27a^T23N^, red indicates the sequence location of dominant negative mutation. (**c**) WT Ad-mCFP-Rab27a was transduced into primary rabbit LGACs (**d**) DN Ad-mCFP-Rab27a was transduced into primary rabbit LGACs. In (**c**,**d**), mCFP was directly imaged with a 458 nm laser. Transduced cells expressing mCFP-Rab27a (green) are outlined in white to delineate the multicellular acinar borders.

In order to study the trafficking of Rab27a in vitro in primary LGAC, we generated monomeric (m) CFP-Rab27a WT and DN adenovirus (Ad) expression constructs. The doxycycline-induced Tet-on Ad construct, shown in Figure 3a, was generated by inserting fragments encoding WT and DN mCFP-Rab27a into a linearized pAdenoX-Tet-3G vector. DN Rab27a, with the T23N mutation shown in Figure 3b, mimics the GDP-bound form of the protein, inhibiting its function. The Ad constructs were characterized by diagnostic enzyme digestion and DNA sequencing (Figure S1) and packaged into 293a helper cells for virus production. High titer Ad were obtained after serial transfection and amplifications, characterized though Western blotting and immunofluorescence of primary transduced mouse LGAC (Figure S1). Western blotting with antibodies to Rab27a and mCFP demonstrated a band of mCFP-Rab27a at ~54 kDa, while the anti-Rab27a antibody also showed a lower band (~27 kDa) representing endogenous Rab27a. Since our functional secretion studies routinely use primary cultured rabbit LGAC, due to the superior cellular yield, we also imaged rabbit LGAC transduced with WT and DN mCFP-Rab27a (MOI = 4–6), (Figure 3c) demonstrating that overexpressed Rab27a had a similar distribution as that observed in LG tissue (Figure 2a) when the CFP was visualized directly. Immunostaining for mCFP in fixed rabbit LGAC also showed vesicle-like structures distributed beneath the apical lumen (Figure S1). Estimates of transduction efficiency in primary rabbit LGAC are estimated as 40–50% (Figure S2). In rabbit LGACs expressing DN mCFP-Rab27a shown in Figure 3d, Rab27a signal was more diffuse and mainly localized in the cytosol.

### 2.4. CCh-Stimulated Rab27a Vesicle Fusion Detected in Primary Cultured Rabbit LGACs

**Figure 4:**
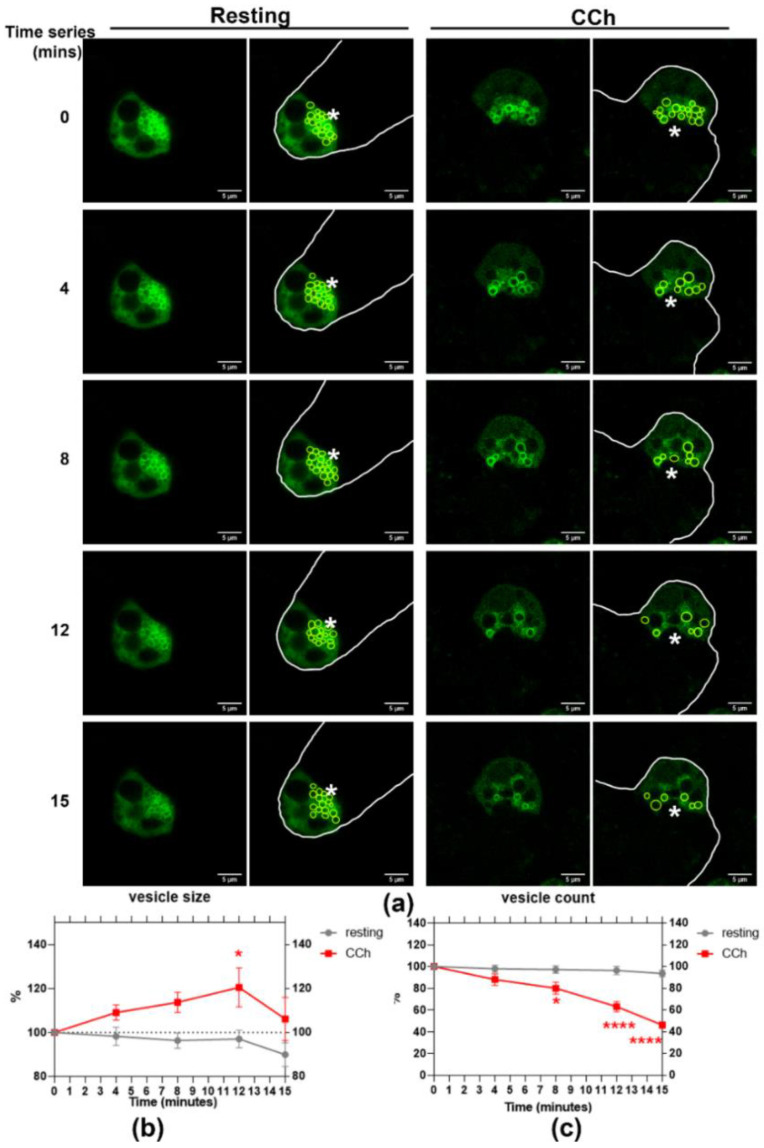
CCh stimulation induces mCFP-Rab27a-enriched vesicle homotypic fusion and vesicle depletion in in primary cultured rabbit LGAC. (**a**) WT Ad-mCFP-Rab27a transduced rabbit LGAC were imaged in time-series experiments before and after CCh stimulation (100 µM). Transduced cells expressing mCFP-Rab27a (green) are outlined in white to delineate cell borders. (**b**) Rab27a-enriched vesicle size was gradually increased upon CCh stimulation, peaking at 12 min (*p* = 0.0182) and then decreasing by 15 min. (**c**) Rab27a-enriched vesicle number was concurrently and significantly decreased by 8 min after stimulation (* *p* = 0.0011), continuing to decrease at 12 and 15 min, **** *p* ≤ 0.0001. No significant changes in these parameters were observed in the resting stage. N = 9, N = cell preparation. Data are presented as mean ± SEM. *, Lumen.

The cholinergic-muscarinic receptor agonist, CCh, stimulates protein and fluid secretion in the LG. To observe the response of Rab27a-enriched vesicles during acinar stimulation using live cell imaging, we transduced primary cultured rabbit LGAC with WT mCFP-Rab27a. Time series live-cell imaging over 15 min was conducted before and after CCh stimulation. Within 4 min of CCh addition, mCFP-Rab27a-enriched vesicles appeared to undergo a homotypic fusion process and then to fuse with the APM, demonstrated by a transiently increased vesicle size followed by reduced vesicle numbers (Figure 4a). The size of mCFP-Rab27a-enriched vesicles was increased upon stimulation, peaked by 12 min of CCh exposure and was then decreased by 15 min (Figure 4b). The number of mCFP-Rab27a-enriched vesicles was decreased with CCh addition, with a significant reduction after 8 min of stimulation (Figure 4c).

### 2.5. Expression of DN Rab27a Significantly Reduces CTSS and β-Hex Secretion in Rabbit LGACs

**Figure 5:**
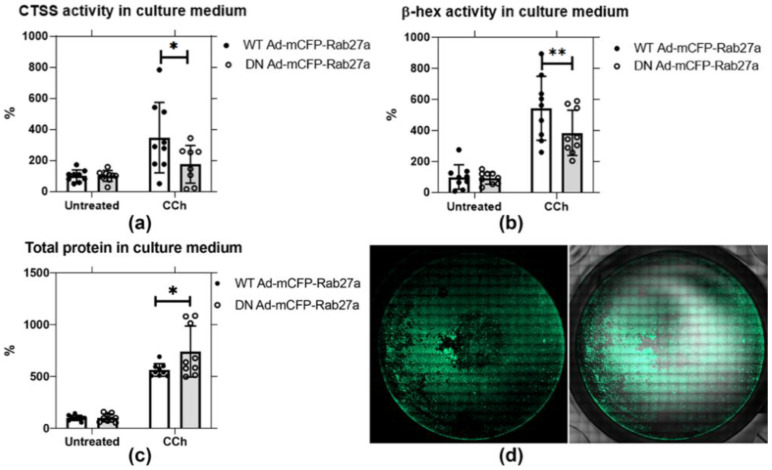
DN Ad-mCFP-Rab27a transduction significantly reduces CTSS and β-hex secretion in primary cultured rabbit LGACs. Secretion assays were conducted in primary rabbit LGACs transduced with adenovirus encoding either mCFP-tagged WT or DN Rab27a Ad at a MOI of 4–6. CTSS and β-hex activity, as well as total protein in cell culture medium were normalized to protein in cell lysate. (**a**) CTSS activity secreted into culture medium with CCh stimulation (100 µM) was significantly reduced by half in cells transduced with the DN mCFP-Rab27a compared to WT Rab27a. *, *p* = 0.0120 (**b**) β-hex activity in culture medium was also significantly reduced by expression of DN mCFP-Rab27a relative to WT mCFP-Rab27a. **, *p* = 0.0020. (**c**) Total protein secreted in response to CCh was significantly increased with DN mCFP-Rab27a relative to WT Rab27a expression. *, *p* = 0.0271. (**d**) LGAC transduction was ~40–50% efficiency, as shown by the representative image for cells transduced with WT mCFP-Rab27a, detailed in Figure S2. N = 3, N= cell preparation. Two to three replicate measurements per preparation, represented as points on the graph, mean ± SD.

Based on their extensive co-enrichment in vesicles in NOD mouse LG (Figure 1), we further probed whether Rab27a might be a principal regulator of CTSS secretion. Analysis of CCh-stimulated secretion conducted in non-transduced rabbit LGAC shows that CCh significantly induces the secretion of CTSS, β-hex, and total protein into culture medium (Figure S3). To test the functional effect of Rab27a, we conducted similar secretion assays in primary cultured rabbit LGAC transduced with WT versus DN mCFP-Rab27a. Rab27a activity, like other Rab proteins, requires the slow hydrolysis of bound GTP to GDP; GTP-bound Rab27a binds to membranes and is considered the activated form: WT Rab27a cycles naturally between GTP and GDP bound states associated with membrane binding and facilitation of trafficking. DN Rab27a^T23N^ sequesters GDP and is trapped in the “inactive state”: its overexpression reduces GTP-bound Rab27a and thus impairs Rab27a activity [48,50]. The LGACs were transduced with WT and DN mCFP-Rab27a at ~40–50% efficiency, demonstrated in Figure 5d and Figure S2. In cells transduced with WT mCFP-Rab27a, as plotted in Figure 5a,b, CTSS and β-hex activity recovered in the cell medium was increased by 3.5-fold and 5-fold upon CCh stimulation relative to their unstimulated controls, respectively. In DN mCFP-Rab27a transduced LGAC, CCh-induced CTSS and β-hex secretion into culture medium was significantly reduced by 0.5-fold and 0.3-fold, compared to secretion from LGAC transduced with the WT. The total protein secreted from DN mCFP-Rab27a transduced cells showed a 20% increase, compared to WT Rab27a transduced cells, as shown in Figure 5c. β-hex is thought to be secreted through the major regulated secretory pathway enriched in Rab3D. The reduction of β-hex secretion in LGACs expressing DN mCFP-Rab27a suggests that Rab27a may participate in this pathway, consistent with its co-enrichment with Rab27b and Rab3D on a subset of apical SV [26]. At the same time, DN mCFP-Rab27a expression more profoundly decreased CTSS activity secreted into culture medium, consistent with our hypothesized role of this protein in CTSS secretion. Increased total protein secreted during Rab27a inhibition may, thus, indicate an upregulation of an alternative secretory pathway.

### 2.6. Endolysosomal Markers, together with Rab27a, Are Redistributed to the APM with Topical CCh Stimulation in Male NOD Mouse LGAC

**Figure 6:**
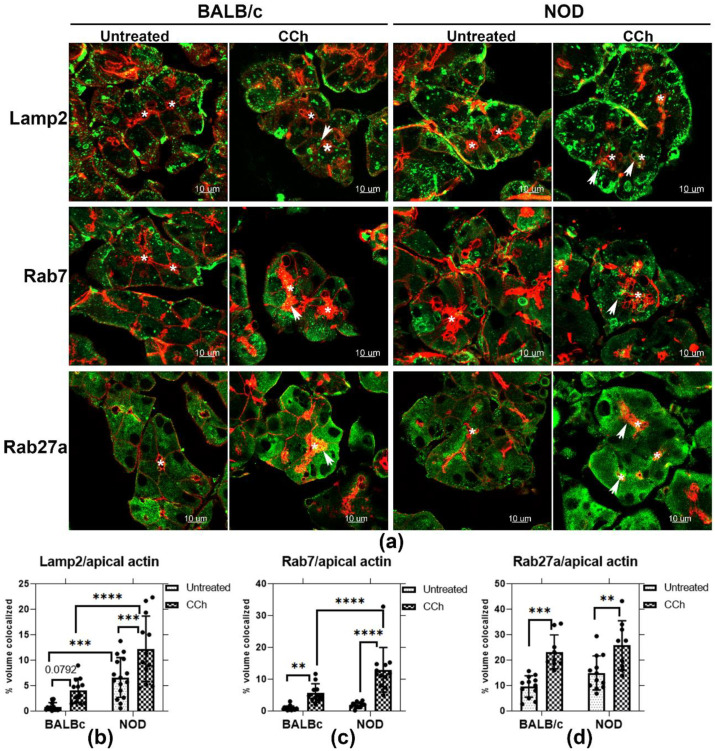
Rab27a and endolysosomal membrane marker colocalization with the apical actin meshwork. (**a**) Enrichment of the endolysosomal markers, Lamp2 and Rab7, as well as Rab27a were evaluated by indirect immunofluorescence in untreated and CCh-stimulated LG from male BALB/c and NOD mice. Z-projection images are shown for Lamp2, Rab7 and Rab27a (green) and the subapical actin meshwork, enriched immediately beneath the APM (red). Colocalization with the apical actin is demonstrated by arrows. (**b**) Lamp2 showed increased accumulation with apical actin in untreated LG of male NOD mouse, compared to LG from BALB/c mouse, with ***, *p* = 0.0003. In stimulated LG, Lamp2 colocalization with the apical actin meshwork increased by 2-fold in LG from male NOD, compared to LG from male BALB/c (****, *p* ≤ 0.0001). Lamp2 colocalization with the apical actin meshwork was increased in both male BALB/c (*p* = 0.0792) and NOD (***, *p* = 0.0007) LG upon stimulation. (**c**) Rab7 colocalization with the apical actin meshwork was increased in both BALB/c (**, *p* = 0.0037) and NOD (****, *p* ≤ 0.0001) mouse LG with stimulation. Stimulated NOD LG sections also showed a 1-fold increase (****, *p* ≤ 0.0001) in colocalization with apical actin, relative to stimulated BALB/c LG sections. (**d**) Rab27a colocalization with the apical actin meshwork was increased in both BALB/c (***, *p* = 0.0001) and NOD (**, *p* = 0.002) mouse LG upon stimulation. However, no obvious differences were observed between the two strains with stimulation. N = 3–4, N = mice per group. Sections from each mouse LG were evaluated in three ROI per sample. All data are presented as mean ± SD. *, Lumen.

Rab27a is implicated in secretion of late endosomes/MVB and lysosomes at the plasma membrane in many cells. To explore the possibility that CCh might trigger direct secretion of Rab27a-enriched endolysosomal membranes at the APM, Z-projection images of Rab27a and the endolysosomal markers, Rab7 and Lamp2, were acquired in BALB/c and NOD LG sections, with and without topical CCh (Figure 6a). Lamp2 is localized primarily on lysosomes and late endosomes [51], while Rab7 is mainly enriched on late endosomes/MVB [52]. Upon topical CCh stimulation, Lamp2, Rab7, and Rab27a in both BALB/c and NOD mouse LGAC were redistributed to the APM region, denoted by the enrichment of the subapical actin meshwork immediately underneath the APM, and as demonstrated by increased colocalization with the apical actin (Figure 6b–d). In unstimulated LG, a significantly increased apical accumulation of Lamp2 was also detected in male NOD mouse LGAC compared to male BALB/c mouse LG. With stimulation, enhanced colocalization of Lamp2 with the apical actin meshwork was observed in NOD LGAC relative to BALB/c LGAC. Similarly, Rab7 was colocalized with the apical actin meshwork with stimulation to a greater extent in NOD mouse LGAC relative to BALB/c mouse LGAC. Rab27a recovery with the apical actin meshwork at the apical lumen was equally enhanced by topical CCh stimulation in both BALB/c and NOD mouse LG. These findings suggest that CCh stimulation may trigger endolysosomal trafficking to the apical membrane area in LGAC, with NOD mouse LGAC demonstrating a more substantial redistribution than BALB/c mouse LGAC. 

### 2.7. Rab27a Distribution on Endolysosomes Is Increased in NOD Mice 

Previously, we reported that CTSS was localized to both endolysosomes and SVs in LG [26]. Upon topical CCh stimulation, tear CTSS activity significantly is increased in NOD mice compared to BALB/c mice. Correlating with the CTSS increased in tears, CTSS protein is depleted in NOD mouse LGAC, as shown in Figure S4, concurrent with detection of some residual CTSS within lumena in stimulated LG tissue in NOD mouse. Density gradient fractionation of membranes from mouse LG showed that CTSS was enriched in a high-density lysosomal membrane fraction along with Rab27a in healthy mouse LG (Figure S5). Moreover, Figure 6 demonstrated the apical redistribution of Rab27a concurrent with the apical accumulation of endolysosomal markers in both male BALB/c and male NOD mouse LG, with a more profound enrichment in NOD mouse LG. These findings collectively suggest Rab27a may mediate trafficking of CTSS between endolysosomes and the APM.

**Figure 7:**
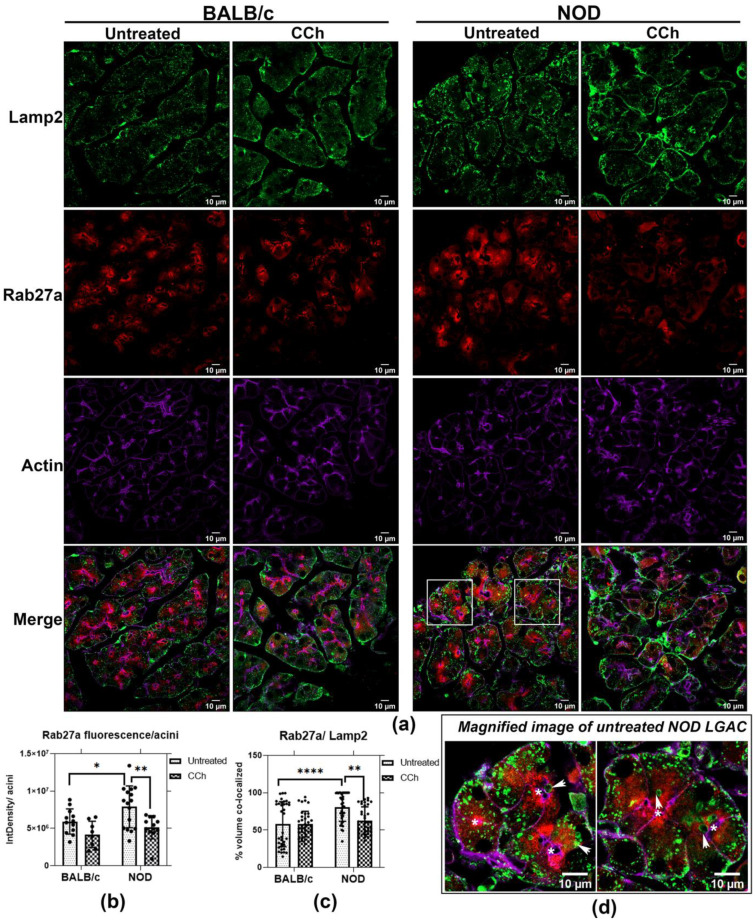
Rab27a enrichment with endolysosomes is increased in LG from male NOD mice. (**a**) Analysis of immunofluorescence by confocal fluorescence microcopy showed that the endolysosomal marker, Lamp2, was partially colocalized with Rab27a in unstimulated acini. (**b**) Upon CCh stimulation, the integrated density of Rab27a within each acinar cell was decreased, with this reduction shown to be significant in NOD mouse LG (*, *p* = 0.0290; **, *p* = 0.0040). Integrated density in three acini were quantifed and average in each field, as described previously [46]. 4 fields were obtained in each mouse section, presented as points. N = 4, N = mouse per group. (**c**) Rab27a colocalization with Lamp2 was significantly increased in unstimulated NOD mouse LG (****, *p* ≤ 0.0001) relative to BALB/c mouse LG, while CCh stimulation significantly reduced this colocalization in NOD mouse LG (**, *p* = 0.0032). (**d**) Magnified images of untreated NOD mouse LGAC demonstrated an accumulation of large Lamp2-enriched vesicles in the apical region, colocalized with Rab27a (White arrows). Colocalization was quantified in three acini per field, four fields were aquired from each mouse section. N = 4, N: mouse per group. *, Lumen.

To further elucidate this relationship, we labeled resting and topically-stimulated LG from BALB/c and NOD mice with antibodies to Rab27a and Lamp2 (Figure 7a). In BALB/c mouse LG, Rab27a was mainly subapically enriched while Lamp2 had a more basolateral distribution. However, an apical accumulation of large Lamp2-enriched vesicles was observed in NOD mouse LG, illustrated in the magnified images of unstimulated NOD mouse LGAC within LG sections (Figure 7), and concurrent with increased colocalization of Rab27a and Lamp2 in unstimulated mouse NOD LG (Figure 7c). With CCh stimulation, a significant reduction in Rab27a and Lamp2 colocalization was observed in NOD mouse LG. This may result from reduced Rab27a disassociation from membranes upon stimulation of secretion, and parallels the loss in mCFP-Rab27a enriched vesicles with CCh stimulation in vitro (Figure 4) and the reduction in integrated density of Rab27a fluorescence/NOD mouse acini, shown in Figure 7b. Z-stack images in Figure 6 and Figure S6 demonstrate that, with stimulation, vesicular Rab27a is both dispersed into the cytoplasm, as well as recruited to the APM region. Decreased association with membranes concurrent with the activation of trafficking pathways has been reported for secretory Rab proteins, consistent with hydrolysis of bound GTP. In Rab27a-mediated exocytosis, after plasma membrane fusion and content release, the GDP dissociation inhibitor is known to translocate GDP-Rab27 from the membrane to the cytosol [48]. These findings suggest a recruitment process occurring simultaneously with exocytosis of Rab27a-enriched endolysosomes upon CCh stimulation. Lamp2, as an integral membrane protein, is present with apical membrane and in retrieved SV membrane following exocytosis, thus remaining enriched at the apical region.

## 3. Discussion

**Figure 8:**
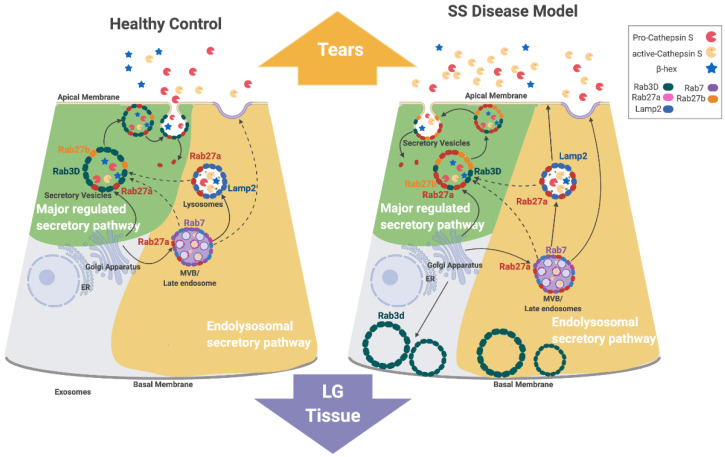
Proposed mechanism of Rab27a involvement in CTSS traffic in LGAC. CTSS is synthesized and initially sorted to endolysosomal compartments. Rab27a regulates CTSS secretion at the APM though both the major regulated secretory pathway and a direct endolysosomal secretory pathway. In healthy LGAC, Rab27a-enriched endolysosomally-derived vesicles may fuse with Rab3D-enriched SV, generating SV enriched in Rab3D, Rab27a and Rab27b containing small amounts of CTSS and other lysosomal enzymes that are secreted with stimulation. In SS, the depletion of Rab3D-enriched SV leads to accumulation of Rab27a-enriched endolysosomally derived vesicles that may accumulate and fuse directly with the APM to release increased CTSS, in the absence of a functional major regulated secretory pathway. Solid lines highlight mechanisms supported as the most likely in the study while dashed arrows represent alternative mechanisms. Figure created with BioRender.com 2020 version (Toronto, ON, Canada).

SS-associated autoimmune dacryoadenitis is linked to elevated CTSS activity in tears and LG, an increase which appears to contribute to ocular inflammation. Concurrent with increased tear CTSS activity, the reduced expression of Rab3D and the redistribution of remaining Rab3D-enriched vesicles is observed in LGAC of both SS patient [28] and male NOD mouse LG [26]. Rab3D and Rab27a/b isoforms are both associated with tear protein exocytosis in LGAC. Previous findings have suggested that reduced Rab3D function contributes to altered tear protein secretion at the APM, leading to changes in tear content in SS. CTSS activity in tears is reduced in mice lacking either Rab27a or b, but enhanced in Rab3D knockout mice [26]. We propose that Rab27a contributes to regulation of CTSS secretion in LGAC and may be a principal driver of the increased tear CTSS secretion seen in the diseased NOD mouse LG. Figure 8 shows the proposed mechanism of Rab27a in CTSS secretion in healthy and diseased (autoimmune dacryoadenitis) LGAC, highlighting its proposed involvement in both the major regulated secretion and a novel endolysosomal secretory pathway that appears to be activated in disease.

In healthy LGAC, we propose that Rab27a mediates the trafficking of endolysosomes to Rab3D-enriched SV. This pathway is responsible for providing low levels of lysosomal enzymes such as CTSS to SV for secretion at the APM into tears. This premise is also consistent with findings by 3D-structured illumination microscopy showing that Rab27a, Rab27b, and Rab3D reside at distinct microdomains on SV in healthy mouse LGAC [26]. This model is further supported by the findings here that inhibition of Rab27a using the DN mCFP-Rab27a construct in vitro impaired the release of β-hex into culture medium from healthy LGAC (Figure 5), reflecting the involvement of Rab27a with Rab3D and Rab27b in formation and fusion of healthy SV containing some lytic enzymes from the major regulated secretory pathway. 

Direct fusion of Rab27a-enriched endolysosomal vesicles with the APM may also occur in healthy LGAC, but appears to be less pronounced than secretion from the major regulated pathway in LGAC when stimulated with CCh. Time lapse imaging experiments in healthy LGAC expressing fluorescently-tagged mCFP-Rab27a showed that CCh could evoke direct exocytosis of mCFP-Rab27a-enriched SV (Figure 4). This fusion appears, however, less vigorous than rates of fusion of fluorescently-labeled Rab3D- and Rab27b-enriched SV with the APM seen in response to CCh stimulation in vitro, suggestive that at least under conditions of engagement of muscarinic receptors, that the direct endolysosomal secretory pathway has a secondary role.

In disease-model male NOD mouse LG, Rab3D expression is decreased, with remaining vesicular stores redistributed to the basolateral area. At the same time, Rab27a-enriched endolysosomal vesicle formation and trafficking of CTSS-enriched vesicles into the apical region is increased. These Rab27a-enriched endolysosomal-derived vesicles may accumulate in the absence of Rab3D-enriched SV, and upon stimulation, undergo homotypic fusion as well as increased direct fusion with the APM. Rab27a’s involvement in increased CTSS secretion in diseased LGAC is supported by findings of increased CTSS colocalized with increased subapical Rab27a-enriched vesicles (Figure 1 and Figure 2) in NOD mouse LGAC which are depleted following CCh (Figure S4). These subapical Rab27a-enriched vesicles are suspected to be primarily secretory vesicles with a few endolysosomes. Our previous work has shown that Rab27a is located on the same vesicles as the traditional secretory vesicle markers, Rab27b and Rab3D [26]. As well, the Rab27a-enriched vesicles range from 0.75–1 µM in diameter, which is the approximate size for secretory vesicles. In Figure 7, an increased colocalization of Rab27a and Lamp2 was observed in the resting LGACs of NOD mouse. These subpically-enriched Rab27a-enriched vesicles in NOD mouse LGAC are also of a reduced size (Figure 2) relative to those in BALB/c mouse LGAC, suggestive of either an impaired terminal maturation process with Rab3D-enriched SV and/or a shift to the endolysosomal secretory pathway involving direct fusion as a dominant secretory pathway. 

A secretagogue-dependent minor regulated secretory pathway has been identified in both pancreatic [37] and parotid acinar cells [53]. In pancreatic acinar cells, Rab27a was shown to mediate this pathway which regulates apical exocytosis of endolysosomes [37]. Primary pancreatic acini isolated from Rab27a-deficient mice exhibited a significant reduction in stimulated amylase release [37]. At the same time, stimulation of WT pancreatic acini recruited Lamp1-enriched vesicles to the APM, while no such effect was observed in pancreatic acini from Rab27a-deficient *ashen* mouse acini, indicating impaired endolysosomal membrane fusion in the absence of Rab27a [37]. We propose that a comparable endolysosomal secretory pathway is responsible for secretion of endolysosomes containing CTSS in healthy LGAC, a process that is dramatically increased in disease. Consistent with this, in otherwise healthy Rab27^ash/ash^Rab27b^−/−^ mouse LG tissue lacking both Rab27 isoform activities, lysosomal accumulation is clearly detectable [40]. Similarly to the studies in pancreatic acini, Lamp2-enriched vesicles colocalized with Rab27a are enriched at the APM region with CCh stimulation, but the enrichment is greater in NOD mouse LG which secrete more CTSS (Figure 6). 

LGAG are innervated by both parasympathetic and sympathetic nerves, which modulate secretion through neurotransmitters and neuropeptides [2]. The interaction of these secretagogues with their receptors serve key roles in activating specific signaling pathways through increased calcium influx and activation of secondary messengers. Our studies have primarily focused on stimulation of muscarinic receptors with CCh. It is possible that engagement of signaling pathways evoked by other neurotransmitters or neuropeptides could more potently activate the endolysosomal secretory pathway, perhaps in response to ocular surface pain or infection, thus triggering the transient release of a more proteolytic tear film. 

The apparent upregulation of the endolysosomal secretory pathway in autoimmune dacryoadenitis may also be associated with alterations in signaling pathways associated with autoimmune inflammation of the LG. Peripheral neuropathy is a key contributor to the pathology of SS [2,54]. The LGAC of MRL/MpJ-Fas^lpr^, another murine model of SS, exhibit a denervation-like supersensitivity to exogenously-added neurotransmitters [55]. This altered sensitivity to secretagogues is thought to result from the presence of proinflammatory cytokines that impair neurotransmitter release from the nerve terminals [55,56]. In aged mice suffering from reduced tear flow, not only was neurotransmitter release defective, but the downstream signaling pathways evoked by cholinergic and α_1_-adrenergic agonists were impaired [57]. The NOD mouse may experience a similarly-impaired neural response in the LG. Altered sensitivity to signaling agonists may contribute to its distinctive secretory features including activation of the endolysosomal secretory pathway. 

We observed what appeared to be larger Rab7-enriched and Lamp2-enriched vesicles beneath the APM in unstimulated NOD mouse acini, relative to unstimulated BALB/c mouse acini, as shown in Figure 6. Imaging of Rab27a and Lamp2 immunofluorescence further demonstrated that these large endolysosomal structures were enriched in Rab27a (Figure 7). These subapical structures may be MVB or amphisomes, which are enriched in both Rab7 and Lamp2 [58], thus suggesting that MVB or amphisome secretion may be increased in diseased LG. Proinflammatory cytokines such as interferon-γ (IFN-γ) and tumor necrosis factor (TNF-α) are highly expressed in NOD mouse LG [27]. LGAC treated in vitro with IFN-γ exhibit characteristics of NOD mouse LGAC including reduced Rab3D [27], increased MHC II [27] and increased CX3CL1 expression [17]. Cultured LGAC exposed to IFN-γ also exhibit enhanced CTSS secretion with CCh stimulation, while β-hex release is unaffected [27]. IFN-γ has been reported to stimulate exosome secretion through Rab27a-mediated release of amphisomes in lung epithelial cells [59]. Studies in eosinophils also reported enhanced MVB membrane fusion and degranulation as well as increased exosome release, with IFN-γ [60]. One factor stimulating the increased secretion of CTSS via Rab27a from NOD mouse LGAC may thus be the local increase in proinflammatory cytokines.

Rab27a was originally thought to be functionally redundant with Rab27b in regulated secretion, since these isoforms share some common effectors [61]. However, Rab27a can play drastically different roles in trafficking relative to Rab27b, depending on the cell type and specific secretory pathways under study, through interaction with distinct effectors [62]. In this study, we did not investigate how Rab27a plays concurrent roles in major and endolysosomal secretory pathways, as our main focus was to address whether Rab27a mediates CTSS secretion in LGAC and how this function is impacted in disease. In future investigations we can explore the interactions of Rab27a with its diverse effectors, and investigate their expression and regulation in disease, to help better explain Rab27a’s ability to participate in different secretory pathways.

In conclusion, Rab27a regulates CTSS secretion though both the major regulated secretory pathway and a novel endolysosomal secretory pathway in LGAC. This study is the first to link Rab27a directly to CTSS secretion in the LG. Our finding is of potential clinical relevance since CTSS is implicated in the etiology of SS [29]. Continuing functional studies and evaluation of protein trafficking in disease-model and healthy mice may in future provide new targets for therapeutic therapeutic development.

## 4. Materials and Methods

### 4.1. Reagents

Carbachol (CCh) (CAS No. 51832), doxycycline monohydrate (CAS no. 17086-28-1) and 4-methylumbelliferyl N-acetyl-β-d-glucosaminide (CAS no. 37067-30-4) were from Sigma-Aldrich (St. Louis, MO, USA). Bovine serum albumin (#2905) was from Calbiochem (Billerica, MA, USA). The Cathepsin S activity kit (#K144) and rabbit polyclonal cathepsin S antibody (#6686) were from Biovision Inc. (Milpitas, CA, USA). Hanks Balanced Salt Solution (HBSS) without Mg^2+^ and Ca^2+^ was from Lonza Group Ltd. (Basel, Switzerland). Rabbit polyclonal anti-Rab27a antibody (#17817-1-AP) was purchased from Proteintech (Rosemont, IL, USA). Mouse monoclonal anti-Rab27a antibody (#ab55667) and rat monoclonal anti-Lamp2 antibody(#ab13524) were from Abcam (Cambridge, MA, USA). Rabbit polyclonal anti-mCFP antibody (#GTX59717) was from GeneTex (Irvine, CA, USA). Rhodamine phalloidin, FITC anti-Rat, Alexa Fluor^®^ 488, 568, 680 secondary antibodies and the ProLong Gold Antifade Mounting Medium were from Invitrogen (Grand Island, NY, USA). Bio-Rad protein assay dye (#5000006) was from Bio-Rad (Hercules, CA, USA). BCA reagents (#23235), CellMask deep red plasma membrane stain (#C10046), 10% Triton X-100 (#NC0478124), 10% tris-glycine gel (#XP0010C), nitrocellulose iBlot™ 2 transfer stacks (#IB23001), TaqMan reverse transcription kit (#N8080234), and primers to mouse *Rab27a* (#Mm00469997) and *Gapdh* (#Mm99999915_g1) were from Thermo Fisher Scientific (Waltham, MA, USA). CloneAmp HiFi PCR Premix (#639298), Adeno-X Adenoviral System 3 (Tet-On 3G Inducible) (#631180), NucleoSpin Gel and PCR Clean-Up kit (#740609), In-Fusion^®^ HD Cloning Kit (#638933), Stella™ competent cells (#63676), Adeno-X screening primer mix 3 (#631030), Terra PCR direct red dye premix (#639286), CalPho mammalian transfection kit (#631312), Adeno-X virus purification kit (#631533) and Adeno-X rapid titer kit (#632250) were from Takara Bio Inc (Mountain View, CA, USA). All restriction enzymes used were purchased from New England Bio Laboratories (Ipswich, MA, USA). Revert™ 700 total protein staining kit (#926-11010) and IRDye^®^ 680RD Donkey anti-Rabbit IgG secondary antibody (#926-68073) were from LI-COR (Lincoln, NE, USA). The QIAquick gel extraction kit (#28704), RNeasy Plus Universal Mini Kit (#73404) and Qiagen plasmid midi kit (#12145) were from Qiagen (Germantown, MD, USA).

### 4.2. Animals

BALB/c (000651) and NOD ShiLtJ (001976) mice were from Jackson Laboratories (Sacramento, CA, USA). All mouse work used LG from male mice aged 16–17 weeks, when lymphocytic infiltration of the LG as well as increased tear CTSS is established [24]. The non-obese diabetic (NOD) mouse is an extensively-used murine model for the autoimmune exocrinopathy characteristic of SS [25]. NOD mice spontaneously develop lymphocytic infiltration of the exocrine glands, resulting in reduced secretory flow [24,25]. The male NOD mouse is more prone to develop ocular manifestations of SS, and to exhibit profound LG infiltration as early as 8–10 weeks of age, while females are more suspectable to infiltration of the SG from 14–16 week of age [17,25]. Mice were anesthetized with a ketamine/xylazine combination at 80–100 mg/kg and 5–10 mg/kg dose, respectively, through intraperitoneal injection. To obtain in situ topically-stimulated glands, mouse LG were surgically-exposed and stimulated topically with carbachol (CCh, 3 µL, 50 µM) for three times, 5 min each, after anesthesia [17]. LG tissues were harvested from mice euthanized by cervical dislocation. Female New Zealand White rabbits (2.1–2.5 kg) were obtained from Western Oregon Rabbit Company, (Philomath, OR, USA). Rabbits were anesthetized through intramuscular injection of ketamine/xylazine at 50 mg/kg and 5 mg/kg doses, respectively. After anesthesia, rabbits were euthanized with intravenous ear injection of 1 mL Euthasol. LG were aseptically dissected from euthanized rabbits and prepared as primary lacrimal acini cultures following published procedures [26]. All animal procedures were conducted in accordance with the Guiding Principles for the Care and Use of Laboratory Animals (8th edition) [63] and approved by the University of Southern California’s Institutional Animal Care and Use Committee.

### 4.3. Tissue Processing and Confocal Fluorescence Microscopy

As previously described [17], LG from mice in which the LG was surgically exposed and treated topically either without and with carbachol (CCh) were retrieved and fixed in 4% paraformaldehyde and 4% sucrose PBS solution for 3 h at room temperature. After fixation, tissues were transferred to 30% sucrose PBS solution and left at 4 °C overnight. LGs were embedding in O.C.T compound and frozen on dry ice. O.C.T blocks were sectioned at 5-µm thickness and mounted on glass micro slides (VWR Superfrost^®^ Plus microslides; VWR, Radnor, PA, USA). The cryosections were quenched with NH4Cl (50 mM) in PBS for 5 min and permeabilized with 0.3% Triton X-100 for 30 min. Slides were then blocked with 5% BSA in 0.3% Triton X-100 for 3 h at room temperature. The tissues were then incubated with primary antibody diluted in blocking buffer at 4 °C overnight. On the second day, the slides were washed in PBS three times, 20 min each, followed by secondary antibody incubation for 1 h at 37 °C. After three more thorough PBS washes, the samples were fixed with ProLong anti-fade mounting medium and imaged the next day. Images were acquired with a Zeiss LSM 800 with Airyscan. Z stack images with a total thickness of 4.96 µm, containing 30 slices at a 0.17-µm interval were acquired. Images were first assigned with random codes to achieve a blinded analysis. Corrected total cell fluorescence quantification was performed using a reported image processing pipeline using ImageJ version 2.1.0 (U. S. National Institutes of Health, Bethesda, MD, USA) [47]. 3D % volume colocalization within regions of interest was carried out using Bitplane Imaris software version 9.0.1 (Andor Technology Ltd., Belfast, UK) according to the manufacturer’s instructions. The % volume colocalization was obtained by quantifying the number of pixels containing both channels above threshold and normalizing to the total number of pixels in the image.

### 4.4. Gene Expression Assay

Mouse LGs were isolated and homogenized in QIAzol lysis buffer with a BeadBlaster 24 microtube homogenizer (Benchmark Scientific, Edison, NJ, USA) at 7 M/S speed for 1 min for two cycles at 30 s intervals. Tissue RNA was extracted with the RNeasy Plus Universal Mini Kit. cDNA was obtained from RNA using the TaqMan reverse transcription kit, as previously described. The reverse transcription reaction was conducted utilizing a GeneAmp PCR System 9700 with incubation cycles of 25 °C (10 min), 48 °C (30 min) and 95 °C (5 min). Quantitative qPCR was carried out with QuantStudio 12K Flex Real-Time PCR System using GAPDH as an internal control. Reaction conditions and calculation methods were as previously described [17,26].

### 4.5. Molecular Cloning of Ad-mCFP-Rab27a Constructs

mCFP-tagged Rab27a plasmid DNA was obtained by subcloning WT mouse Rab27a (678bp) and the DN mutant (Rab27a^T23N^) into vector pCMV6-AN-mCFP (#6631, Takara Bio Inc.) at the Hind III-Mlu I cloning site. Plasmid synthesis and subcloning were carried out by Genescript (Piscataway, NJ) gene services. The plasmid insert mCFP-Rab27a was amplified through RT-PCR with forward primer, 5′-GTAACTATAACGGTCATGAGCGGGGGCGAGGAG-3′ and reverse primer 5′ATTACCTCTTTCTCCTTAACGCGTTCAACAGCCACAC-3′ using CloneAmp HiFi PCR Premix according to the company’s instructions. Agarose gel analysis and the NucleoSpin gel PCR clean up kit were used to analysis and purify the PCR inserts. The purified WT and DN mCFP-Rab27a were then inserted into a linearized pAdenoX-Tet3G vector though in-fusion cloning by incubation with 5× in-fusion HD enzyme premix for 15 min at 50 °C. The recombinant adenoviral constructs were transformed in Stella™ competent cells. 1.5 µL in-fusion reactions were added to 100 µL Stella stock, incubated on ice for 30 min and heat-shocked for 45 s at 42 °C. A total of 900 µL of the SOC medium was added to the cell mix and shaken at 37 °C for 1 h. 100 µL from each transformation was plated onto LB agar plates containing 100 µg/mL ampicillin. Well-separated colonies were picked from the plates and screened by RT-PCR with Adeno-X screening primer mix 3 and Terra PCR direct red dye premix. After the recommended number of thermal cycles, 5 µL of each PCR reaction was analyzed by 1.2% agarose gel. The positive clones were amplified by inoculating 100 mL of liquid LB/Ampicillin Medium (100 μg/mL ampicillin) with 2–5 mL of fresh, log phase culture and shaken overnight at 37 °C at 300 rpm for 12–16 h. The amplified bacteria were then lysed and plasmid DNA purified with the Qiagen plasmid midi kit according to kit instructions. Plasmid quality was evaluated through individual digestions with Xho I and Nhe I and analysis on a 1.2% agarose gel. Bands containing mCFP-tagged WT and DN Rab27a fragments were excised and plasmid fragments extracted using the QIAquick Gel Extraction Kit. The extracted fragments were sent to Retrogen Inc (San Diego, CA, USA). for Sanger sequencing. After confirming the sequence for both mCFP-tagged WT and DN Rab27a, the adenovirus plasmids were linearized by incubating with PacI restriction enzyme at 37 °C for 2 h to expose inverted terminal repeats. The digestion process was monitored using a 1.2% agarose gel to monitor the migration of the resulting 3 kb fragment with completion. 60 μL 1 × TE Buffer (pH 8.0) and 100 μL phenol: chloroform: isoamyl alcohol (25:24:1) were added to the linearized plasmids with gentle vortexing. The mixtures were then centrifuged at 14,000 rpm for 5 min at 4 °C to separate phases. The top aqueous layer was removed and added to a mixture of 400 μL 95% ethanol, 25 μL 10 M NH_4_OAc (or 1/10 volume 3 M NaOAc), and 1 μL glycogen (20 mg/mL). The mixture was centrifuged at 14,000 rpm at 4 °C for 5 min, and the pellets collected, washed with 300 μL 70% ethanol, and redissolved in 10 μL sterile 1 × TE Buffer (pH 8.0).

### 4.6. Adenovirus Production and Amplification

QBI-HEK 293 cells (R70507) were obtained from Qbiogene, Adenovirus Technology (Carlsbad, CA, USA) and cultured in high glucose DMEM supplemented with 10% FBS, 0.1 mM MEM non-Essential amino acids (NEAA), 2 mM L-glutamine and 1% Pen-Strep. 293A cells were plated on a 60 mm culture plate 24 h before transfection. Once cells reached 60% confluency, PacI-digested adenovirus DNAs encoding WT and DN mCFP-Rab27a were added using the CalPho mammalian transfection kit according to the manufacturer’s manual. The cells started to display cytopathic effects after 2–3 days. After one week in culture, the cells were harvested and lysed through three consecutive freeze-thaw cycles. The lysates were utilized to further infect a fresh 60-mm culture plate of cells with cells collected, lysed after development of cytopathic effects, and the supernatant used to infect larger cultures. Ad-transfected cells were harvested and purified with the Adeno-X virus purification kit according to the kit instructions. Adenovirus titer was measured by both plaque assay as previously reported [26] and with the Adeno-X rapid titer kit.

### 4.7. Rabbit LGAC Preparation and Transfection

Isolation of rabbit LGACs was as previously described [26,40]. At day 1 after isolation, LGACs were transduced with high titer adenovirus. Cells were incubated with mCFP-tagged WT or DN Rab27a adenovirus for 4 h at a multiplicity of infection (MOI) of 4–6. Medium containing the Ad was removed and replaced with fresh Peter’s complete medium (PCM) containing 1 µg/mL doxycycline to induce protein expression. Cells were cultured for another 18–20 h for optimal protein expression. LGAC were kept in culture for a total of two days prior to functional secretion and imaging experiments. During culture, cells aggregate into acinus-like structures, where distinct apical and basolateral domains are presented, mimicking the structures and functions of acini in vivo [26,40].

### 4.8. Live Cell Imaging of Rabbit LGACs

LGACs were seeded in 35-mm glass bottomed petri dishes coated with Matrigel at a cell density of 6 × 10^6^ cells per dish. After transduction with WT mCFP-Rab27a and incubation with PCM containing 1 µg/mL doxycycline, the medium was gently removed and replaced with Mg^2+^-free HBSS. During time lapse imaging, images of single cell focal planes were acquired for 15 min at a fixed time interval of 10 s. Resting state videos were recorded by imaging cells in a 37 °C imaging chamber for 15 min. After acquisition of these videos, 100 μM CCh in Mg^2+^-free HBSS was added to the cells to stimulated regulated exocytosis and imaged for another 15 min. Similarly, the same volume of Mg^2+^-free HBSS was added to the cells and used as an unstimulated blank control. Vesicle sizes and counts were quantified by Image J. Images were acquired with a Zeiss LSM880 using the 458 nm laser line.

### 4.9. In Vitro Secretion of Primary Rabbit LGAC

As previously described [26], LGACs were seeded in 12-well plates coated with Matrigel at a cell density of 2 × 10^6^ per well. Cells were transduced as described above. Prior to secretion assays, cells were pre-incubated in Mg^2+^-free HBSS at 37 °C for 1 h and the medium collected as the basal secretion control. Cells were then treated with CCh (100 μM) in Mg^2+^-free HBSS at 37 °C for 30 min to stimulate secretion. The same volume of medium was added into the cells under the same conditions for the untreated controls. The cell culture medium was collected post-incubation and the cells lysed with NaOH. CTSS activity in the cell culture medium was measured using the CTSS activity fluorometric assay kit according to the manufacturer’s instructions at 37 °C for 2 h. Fluorescence was measured using 400/505 nm excitation/emission filters. β-hex activity was measured as previously described [26], with incubation of the reaction mixture at room temperature for 2 h and measurement of signal using 365/460-nm excitation/emission filters in a SpectraMax iD3 (Molecular Devices, San Jose, CA, USA). Total protein in culture medium was measured by the Bio-Rad assay protein assay. The total protein in cell lysates was measured using the Pierce BCA Protein Assay (Thermo Fisher Scientific, Waltham, MA, USA, #23225) as previously reported. Activities of CTSS and β-hex, together with media protein, were normalized to total cell protein in each well, as relative fluorescence per microgram protein.

### 4.10. Statistics

All statistical analyses were performed using Graphpad Prism 8.3.0 software (San Diego, CA, USA). Data normality was assessed by Kolmogorov-Smirnov, D’Agostino and Person omnibus, and Shapiro–Wilk normality tests. A two-tailed unpaired Student’s *t*-test was used to compare between two treatment groups. A two-way ANOVA with Sidak’s multiple comparison test were used to compare different treatment effects between two groups. The criterion for statistical significance was set at *p* ≤ 0.05. In all graphs, ns, *p* > 0.05; * *p* ≤ 0.05; **, *p* ≤ 0.01; ***, *p* ≤ 0.001; ****, *p* ≤ 0.0001.

## Data Availability

The data that support the findings of this study are available from the corresponding author upon reasonable request.

## Supplementary Materials

The following are available online at https://www.mdpi.com/1422-0067/22/4/1630/s1.

## Abbreviations

ADDE	Aqueous-Deficient Dry Eye
APM	Apical Plasma Membrane
β-hex	β-Hexosaminidase
CCh	Carbachol
CTSS	Cathepsin S
DN	Dominant negative
IFN-γ	Interferon-γ
LG	Lacrimal Gland
LGAC	Lacrimal Gland Acinar Cells
MVB	Multivesicular Bodies
NOD	Non-obese Diabetic
PCM	Peter’s complete medium
SS	Sjögren’s syndrome
SG	Salivary Gland
SV	Secretory Vesicle
TNF-α	Tumor Necrosis Factor
WT	Wild-type

## References

[1] Edman M.C., Marchelletta R.R., Dartt D.A. (2010). Lacrimal Gland Overview. Encyclopedia of the Eye.

[2] Dartt D.A. (2009). Neural Regulation of Lacrimal Gland Secretory Processes: Relevance in Dry Eye Diseases. Prog. Retin. Eye Res..

[3] Pflugfelder S.C., de Paiva C.S. (2017). The Pathophysiology of Dry Eye Disease: What We Know and Future Directions for Research. Ophthalmology.

[4] Brito-Zerón P., Baldini C. (2016). Sjögren syndrome. Nat. Rev. Dis. Primers.

[5] Nocturne G., Mariette X. (2015). Sjögren Syndrome-associated lymphomas: An update on pathogenesis and management. Br. J. Haematol..

[6] Hyon J.Y., Lee Y.J. (2007). Management of Ocular Surface Inflammation in Sjögren Syndrome. Cornea.

[7] Stern M.E., Pflugfelder S.C. (2004). Inflammation in Dry Eye. Ocul. Surf..

[8] Chen X., Aqrawi L.A. (2019). Elevated cytokine levels in tears and saliva of patients with primary Sjögren’s syndrome correlate with clinical ocular and oral manifestations. Sci. Rep..

[9] Na K.-S., Mok J.-W. (2012). Correlations between Tear Cytokines, Chemokines, and Soluble Receptors and Clinical Severity of Dry Eye Disease. Investig. Ophthalmol. Vis. Sci..

[10] Byun Y.-S., Lee H.J. (2017). Elevation of autophagy markers in Sjögren syndrome dry eye. Sci. Rep..

[11] Dartt D.A. (2004). Dysfunctional Neural Regulation of Lacrimal Gland Secretion and its Role in the Pathogenesis of Dry Eye Syndromes. Ocul. Surf..

[12] Mavragani C.P., Moutsopoulos H.M. (2014). Sjögren’s Syndrome. Annu. Rev. Path Mech. Dis..

[13] Foulks G.N., Forstot S.L. (2015). Clinical Guidelines for Management of Dry Eye Associated with Sjögren Disease. Ocul. Surf..

[14] Reiser J., Adair B. (2010). Specialized roles for cysteine cathepsins in health and disease. J. Clin. Investig..

[15] Shi G.-P., Villadangos J.A. (1999). Cathepsin S Required for Normal MHC Class II Peptide Loading and Germinal Center Development. Immunity.

[16] Kirschke H., Rawlings N.D., Salvesen G. (2013). Chapter 413-Cathepsin S. Handbook of Proteolytic Enzymes.

[17] Fu R., Guo H. (2020). Cathepsin S activation contributes to elevated CX3CL1 (fractalkine) levels in tears of a Sjögren’s syndrome murine model. Sci. Rep..

[18] Klinngam W., Fu R. (2018). Cathepsin S Alters the Expression of Pro-Inflammatory Cytokines and MMP-9, Partially through Protease—Activated Receptor-2, in Human Corneal Epithelial Cells. Int. J. Mol. Sci..

[19] Hou W.-S., Li W. (2002). Comparison of cathepsins K and S expression within the rheumatoid and osteoarthritic synovium. Arthritis Rheumatol..

[20] Kim S.J., Schätzle S. (2017). Increased cathepsin S in Prdm1−/− dendritic cells alters the TFH cell repertoire and contributes to lupus. Nat. Immunol..

[21] Haves-Zburof D., Paperna T. (2011). Cathepsins and their endogenous inhibitors cystatins: Expression and modulation in multiple sclerosis. J. Cell. Mol. Med..

[22] Richard D.A.W., Williams R. (2015). Cathepsin S: Therapeutic, diagnostic, and prognostic potential. In Biol. Chem..

[23] Hamm-Alvarez S.F., Janga S.R. (2014). Tear Cathepsin S as a Candidate Biomarker for Sjögren’s Syndrome. Arthritis Rheumatol..

[24] Janga S.R., Shah M. (2019). Longitudinal analysis of tear cathepsin S activity levels in male non-obese diabetic mice suggests its potential as an early stage biomarker of Sjögren’s Syndrome. Biomarkers.

[25] Ju Y., Janga S.R. (2018). NOD and NOR mice exhibit comparable development of lacrimal gland secretory dysfunction but NOD mice have more severe autoimmune dacryoadenitis. Exp. Eye Res..

[26] Meng Z., Edman M.C. (2016). Imbalanced Rab3D versus Rab27 increases cathepsin S secretion from lacrimal acini in a mouse model of Sjögren’s Syndrome. Am. J. Physiol. Cell Physiol..

[27] Meng Z., Klinngam W. (2017). Interferon-γ treatment in vitro elicits some of the changes in cathepsin S and antigen presentation characteristic of lacrimal glands and corneas from the NOD mouse model of Sjögren’s Syndrome. PLoS ONE.

[28] Bahamondes V., Albornoz A., Aguilera S. (2011). Changes in Rab3D expression and distribution in the acini of Sjögren’s syndrome patients are associated with loss of cell polarity and secretory dysfunction. Arthritis Rheumatol..

[29] Klinngam W., Janga S.R. (2019). Inhibition of Cathepsin S Reduces Lacrimal Gland Inflammation and Increases Tear Flow in a Mouse Model of Sjögren’s Syndrome. Sci. Rep..

[30] Deneka M., Neeft M. (2003). Regulation of Membrane Transport by rab GTPases. Crit. Rev. Biochem. Mol. Biol..

[31] Deacon S.W., Gelfand V.I. (2001). Of Yeast, Mice, and Men: Rab Proteins and Organelle Transport. J. Cell Biol..

[32] Izumi T., Gomi H. (2003). The Roles of Rab27 and Its Effectors in the Regulated Secretory Pathways. Cell Struct. Funct..

[33] Schlüter O.M., Khvotchev M. (2002). Localization Versus Function of Rab3 Proteins: Evidence for a Common Regulatory Role in Controlling Fusion. J. Biol. Chem..

[34] Riedel D., Antonin W. (2002). Rab3D Is Not Required for Exocrine Exocytosis but for Maintenance of Normally Sized Secretory Granules. Mol. Cell. Biol..

[35] Chen D., Guo J. (1997). Molecular Cloning and Characterization of Rab27a and Rab27b, Novel Human Rab Proteins Shared by Melanocytes and Platelets. Exp. Mol. Med..

[36] Chen X., Li C. (2004). Rab27b localizes to zymogen granules and regulates pancreatic acinar exocytosis. Biochem. Biophys. Res. Commun..

[37] Hou Y., Ernst S.A., Stuenkel E.L. (2015). Rab27A Is Present in Mouse Pancreatic Acinar Cells and Is Required for Digestive Enzyme Secretion. PLoS ONE.

[38] Yi Z., Yokota H. (2002). The Rab27a/Granuphilin Complex Regulates the Exocytosis of Insulin-Containing Dense-Core Granules. Mol. Cell. Biol..

[39] Imai A., Yoshie S. (2004). The small GTPase Rab27B regulates amylase release from rat parotid acinar cells. J. Cell Sci..

[40] Chiang L., Ngo J. (2011). Rab27b regulates exocytosis of secretory vesicles in acinar epithelial cells from the lacrimal gland. Am. J. Physiol. Cell Physiol..

[41] Tolmachova T., Anders R., Stinchcombe J. (2003). A General Role for Rab27a in Secretory Cells. Mol. Biol. Cell.

[42] Pfeffer S.R. (2010). Two Rabs for exosome release. Nat. Cell Biol..

[43] Ostrowski M., Carmo N.B. (2010). Rab27a and Rab27b control different steps of the exosome secretion pathway. Nat. Cell Biol..

[44] Van der Sluijs P., Zibouche M. (2013). Late Steps in Secretory Lysosome Exocytosis in Cytotoxic Lymphocytes. Front. Immunol..

[45] Neeft M., Wieffer M. (2004). Munc13-4 Is an Effector of Rab27a and Controls Secretion of Lysosomes in Hematopoietic Cells. Mol. Biol. Cell.

[46] Tyrpak D. (2019). Corrected-Total-Cell-Fluorescence.

[47] Tyrpak D., Li Y. (2020). SIAL: A simple image analysis library for wet-lab scientists. J. Open Source Softw..

[48] Imai A., Tsujimura M. (2017). The small GTPase, Rab27, and its effectors and regulators participate in granule exocytosis by parotid acinar cells. J. Oral Biosci..

[49] Hammel I., Lagunoff D. (2010). Regulation of secretory granule size by the precise generation and fusion of unit granules. J. Cell Mol. Med..

[50] Yamaoka M., Ishizaki T. (2015). GTP- and GDP-Dependent Rab27a Effectors in Pancreatic Beta-Cells. Biol. Pharm. Bull..

[51] Eskelinen E.-L., Illert A.L. (2002). Role of LAMP-2 in lysosome biogenesis and autophagy. Mol. Biol. Cell.

[52] Guerra F., Bucci C. (2016). Multiple Roles of the Small GTPase Rab7. Cells.

[53] Castle A.M., Huang A.Y. (2002). The minor regulated pathway, a rapid component of salivary secretion, may provide docking/fusion sites for granule exocytosis at the apical surface of acinar cells. J. Cell Sci..

[54] Hocevar A., Tomsic M. (2003). Parasympathetic nervous system dysfunction in primary Sjögren’s syndrome. Ann. Rheum. Dis..

[55] Zoukhri D., Kublin C.L. (2001). Impaired neurotransmitter release from lacrimal and salivary gland nerves of a murine model of Sjögren’s syndrome. Investig. Ophthalmol. Vis. Sci..

[56] Zoukhri D., Hodges R.R. (2002). Role of proinflammatory cytokines in the impaired lacrimation associated with autoimmune xerophthalmia. Investig. Ophthalmol. Vis. Sci..

[57] Ríos J.D., Horikawa Y., Chen L.-L., Kublin C.L., Hodges R.R., Dartt D.A., Zoukhri D. (2005). Age-dependent alterations in mouse exorbital lacrimal gland structure, innervation and secretory response. Exp. Eye Res..

[58] Hanson P.I., Cashikar A. (2012). Multivesicular Body Morphogenesis. Annu. Rev. Cell Dev. Biol..

[59] Chen Y.-D., Fang Y.-T. (2017). Exophagy of annexin A2 via RAB11, RAB8A and RAB27A in IFN-γ-stimulated lung epithelial cells. Sci. Rep..

[60] Mazzeo C., Cañas J.A. (2015). Exosome secretion by eosinophils: A possible role in asthma pathogenesis. J. Allergy Clin. Immunol..

[61] Barral D.C., Ramalho J.S. (2002). Functional redundancy of Rab27 proteins and the pathogenesis of Griscelli syndrome. J. Clin. Investig..

[62] Fukuda M. (2013). Rab27 Effectors, Pleiotropic Regulators in Secretory Pathways. Traffic.

[63] Garber J.C., Barbee R.W. (2011). Guide for the Care and Use of Laboratory Animals.

