# Supplementary material for: Rab27a Contributes to Cathepsin S Secretion in Lacrimal Gland Acinar Cells
Source: Int J Mol Sci. 2021 Feb 5;22(4):1630. doi: 10.3390/ijms22041630 (PMC7914720; doi:10.3390/ijms22041630)
Supplement: Supplementary file 1 [file ijms-22-01630-s001.pdf]

Supplementary Materials

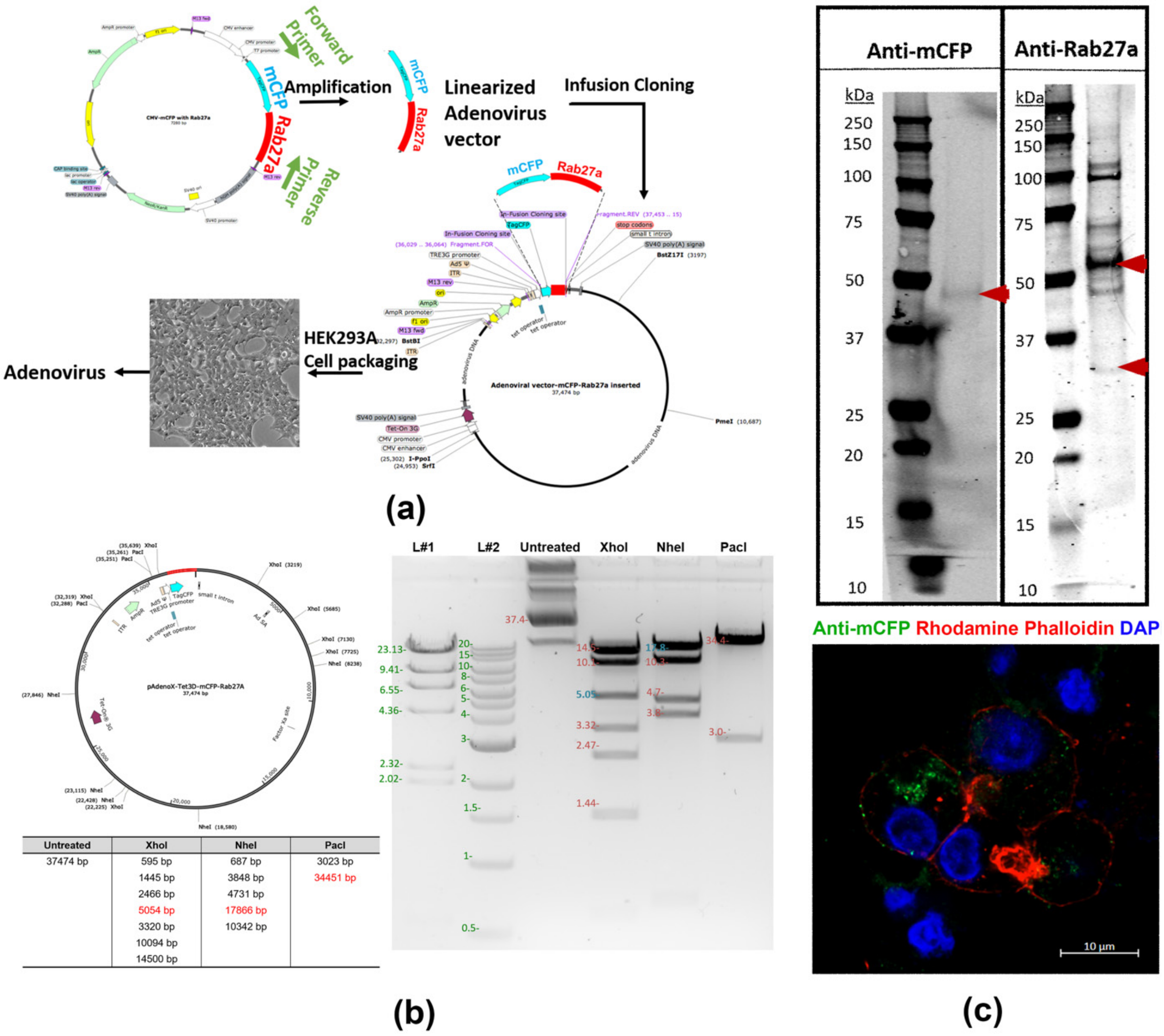

Untreated	XhoI	NheI	PacI
37474 bp	595 bp	687 bp	3023 bp
	1445 bp	3848 bp	34451 bp
	2466 bp	4731 bp	
	5054 bp	17866 bp	
	3320 bp	10342 bp	
	10094 bp		
	14500 bp		

Figure S1. Ad-mCFP-Rab27a production and plasmid validation**. (a)** WT and DN Ad-mCFP-Rab27a were generated by infusion cloning of the respective mCFP-Rab27a inserts into a Tet-on Ad vector, which was further packaged into 293a helper cells for virus production. **(b)** Prior to 293a cell transduction, the WT Ad-mCFP-Rab27a construct was verified by diagnostic enzyme digestion insert sequencing. L#1-2 are loading ladders. The DN Ad-mCFP-Rab27a construct was similarly verified **(c)** WT mCFP-Rab27a protein expression was characterized by Western blotting and immunofluorescence microscopy in primary cultured mouse LGAC from C57BL/6 mouse LG. Both anti-mCFP and anti-Rab27a antibodies labeled a CFP-tagged Rab27a of ~54kDa. c) By immunofluorescence microscopy, CFP labeled by anti-mCFP antibody is shown in green, actin cytoskeleton labeled by rhodamine phalloidin is shown in red, and nuclei labeled by DAPI is in blue.

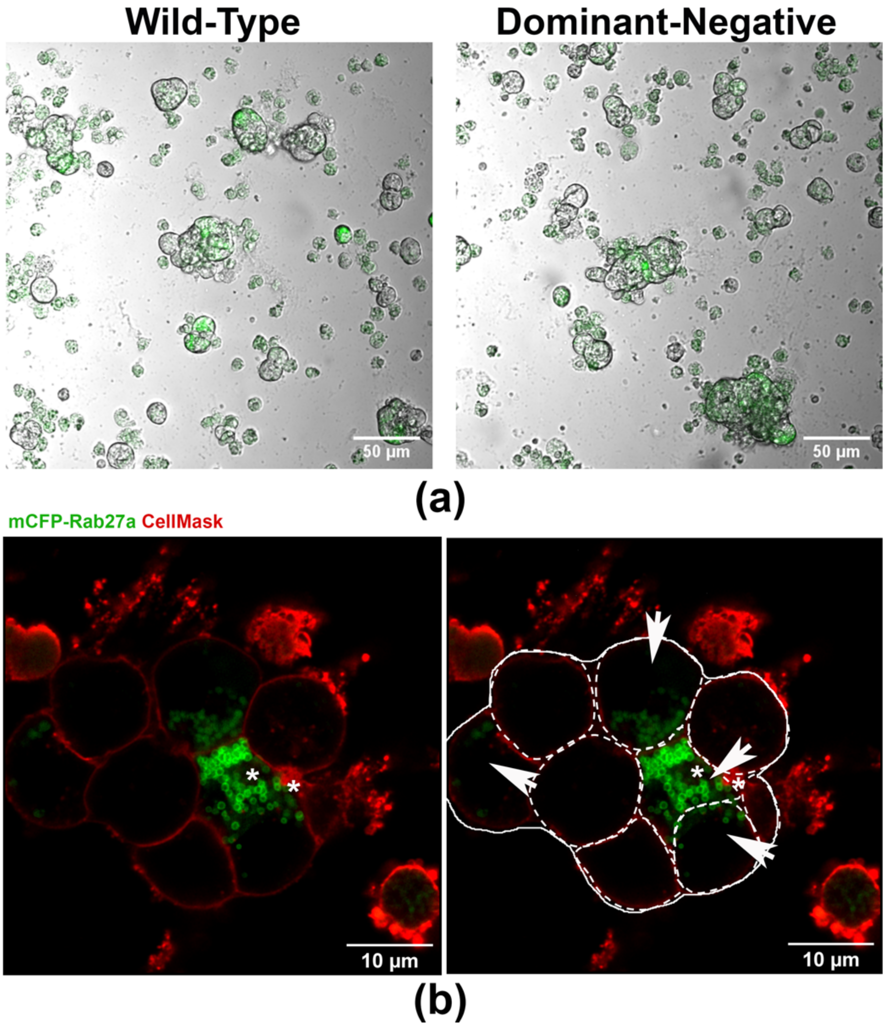

Figure S2. Estimated transduction efficiency for Ad-mCFP-Rab27a transduction in rabbit LGACs. Based on the 3D structures of acini clusters, we estimated transduction efficiency through phase imaging of acinar clusters and evaluating transduction efficiency of individual cells in the cluster. (**a**) Phase imaging (20X) of primary rabbit lacrimal gland acinar cells transduced with wild-type and dominant-negative Ad-mCFP-Rab27a (MOI of 4~6). 60~80% of the acinar clusters were transduced. (**b**) Representative image of transduction efficiency quantification within each acinar cluster. Cell membranes were labeled with CellMask plasma membrane stain (red). The acinar borders are outlined by a solid white line, while individual cells within the acinar cluster are outlined with dashed white lines. Arrowheads show the cells with fluorescence signal. *, Lumen.

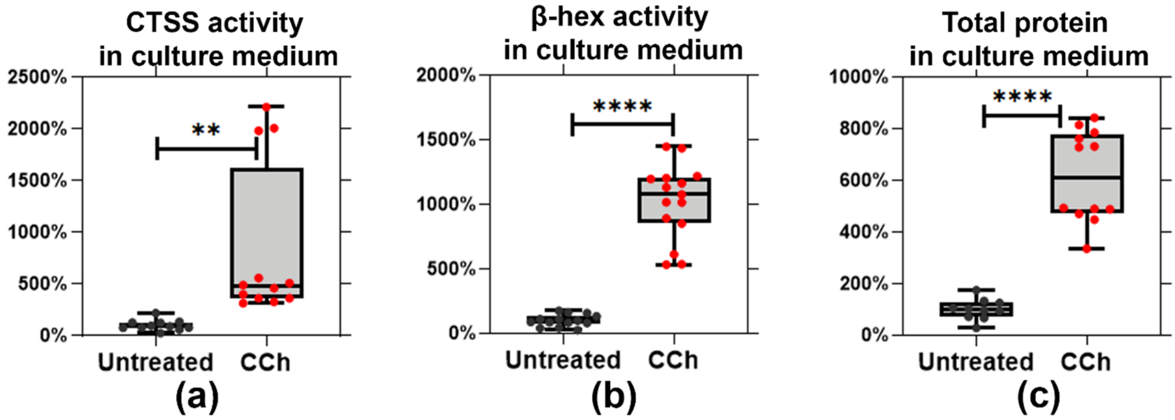

Figure S3. CCh stimulation induces CTSS, β-hex and total protein secretion from primary cultured rabbit LGACs. CTSS, β-hex and total protein in cell culture medium without and with CCh (100 μM) were normalized to cell protein in each well. (**a**) CTSS activity in culture medium was increased by 5-fold, with $p = 0.0041$; (**b**) β-hex activity in culture medium was increased by 10-fold, $p \leq 0.0001$ and (**c**) total protein in culture medium was increased by 6-fold, $p \leq 0.0001$, compared to the unstimulated controls. N = 5, N: cell preparation. 2~3 replicate measurements per preparation, represented as points on graph, mean ± SD.

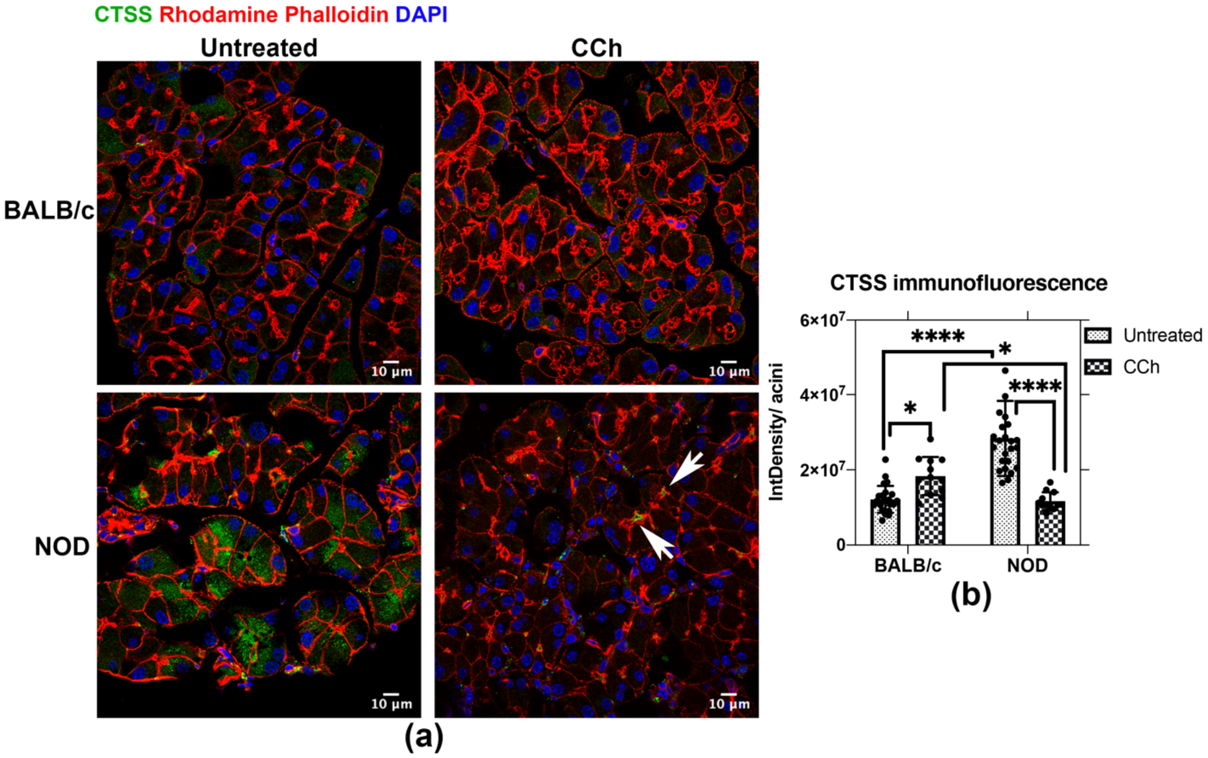

Figure S4. CTSS immunofluorescence is significantly reduced by topical CCh stimulation in NOD mouse relative to BALB/c mouse LG. CTSS Integrated density was quantified using the CTCF method[1, 2] in each acini, with 5 acini quantified per field, 5 fields were obtained from each mouse LG. (**a**) CTSS immunofluorescence is increased in the subapical area in NOD mouse LG without stimulation. Topical CCh stimulation depletes the subapical CTSS in the acini, with residual protein detected within the lumen (arrowheads). CTSS labeling by immunofluorescence is in green, actin cytoskeleton labeling by rhodamine phalloidin is shown in r ed, while nuclear labeling by DAPI is shown in blue. (**b**) Quantification of (**a**) showed that CTSS is significantly increased in unstimulated male NOD mouse LGAC ($p \leq 0.0001$), compared to BALB/c mouse LG. CCh stimulation significantly reduced the CTSS recovered in NOD mouse LGAC by 60% ($p \leq 0.0001$). N = 3-5, N: mouse per group. Average integrated density per acini in each field were presented as points.

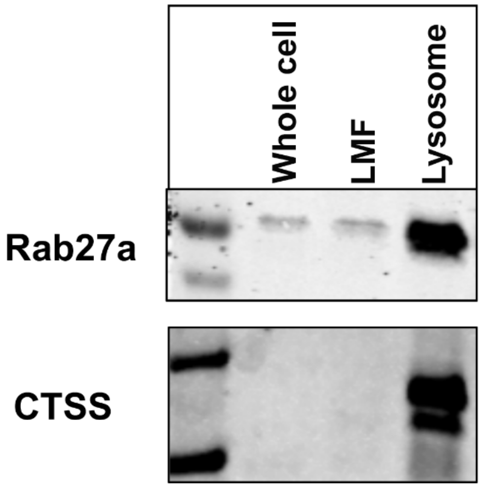

Whole cell: clarified cell lysate
LMF:Light mitochondrial fraction
Lysosome: Lysosomal fraction

Figure S5. Rab27a is co-enriched with lysosomal fractions containing CTSS. Clarified cell lysate, the light mitochondrial fraction (LMF) and the lysosomal fraction were prepared from C57BL/6 mouse LGs using an Optiprep density gradient [3]. Each lane was loaded with an equal amount of protein (35 μg/lane). Rab27a , detected by Western blotting, was enriched in the lysosomal fraction, compared to whole cell lysate or LMF. An accumulation of CTSS was also detected in the Rab27a-enriched lysosomal fraction.

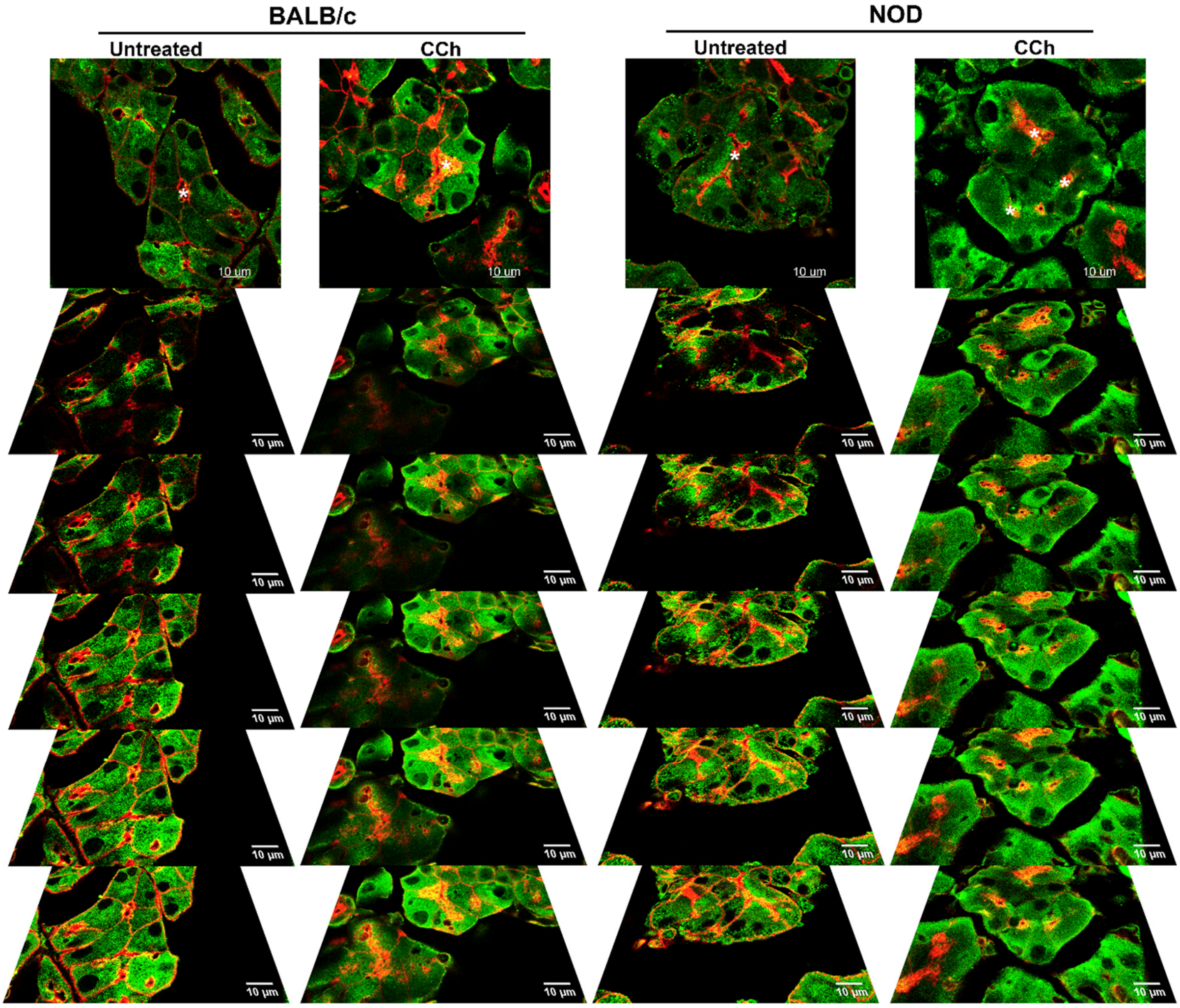

Figure S6. Vesicular Rab27a is dispersed into the cytoplasm in CCh-stimulated LG. Z-stack images of resting and CCh-stimulated LGACs show that, in resting acini, Rab27a is localized to vesicles enriched in the subapical area from both BALB/c and NOD mice, but NOD mouse LGAC exhibit more accumulation near the lumen (asterisk). After CCh stimulation, Rab27a is dispersed into the cytoplasm as well as recruited to the APM in both BALB/c and NOD.

Supplementary Methods

Lysosome Density Gradient Isolation and Western Blotting

LGs from 5 mice were harvested and homogenized with a Dounce homogenizer in homogenization medium (HM, 0.25 M sucrose, 1 mM Na_2EDTA, 10 mM HEPES, pH = 7). Homogenates were centrifuged for 10 min at 750× *g* at 4 °C. The supernatant (clarified cell lysate) was collected and centrifuged for 10 min at 20,000× *g* at 4 °C. The pellet was collected and resuspended in 3.5 mL homogenization medium, identified as the light mitochondrial fraction (LMF). The lysosomal fraction was obtained through separation using an Opti-prep density gradient [3]. 50% (*w*/*v*) iodixanol stock was prepared by diluting OptiPrep (60% iodixanol) into HM medium. A linear OptiPrep gradient was prepared by overlaying 6 mL of 19% (*w*/*v*) iodixanol and 6 mL of 27% (*w*/*v*) iodixanol in homogenization medium. 50 nM LysoTracker Deep Red was added to the LMF fraction for band visualization during collection. LMF was loaded at the bottom of the gradient with a syringe and centrifuged for 2 h at 110,000× *g* at 4 °C. The lysosomal band, which was close to the top of the gradient and identified by its red fluorescence, was carefully collected with a syringe. The lysosomal sample was diluted in 12 mL of HM medium and centrifuged again for 30 min at 30,000× *g* at 4 °C. The pellet

(lysosomal fraction) was resuspended in 0.5 mL HM medium for Western Blot analysis. The protein concentration in each fraction was analyzed by the BCA assay. The samples were left in reducing Laemmli-SDS sample buffer dye for 2 h at 4 °C. 35 μg of sample was loaded into each well of a precast 10% Tris-Glycine gel and resolved under constant voltage (80 V) at 4 °C for 2 h. The proteins were then transferred to a nitrocellulose membrane using the iBlot 2 dry blotting system (Thermo Fisher Scientific, Waltham, MA, USA). The membrane was blocked with Rockland fluorescent blocking buffer for 1 h at room temperature and incubated overnight with primary Rab27a antibody (1:1000 dilution in blocking buffer) at 4 °C. On the second day, the membrane was washed with 1X Tris-Buffered Saline with 0.1% Tween® 20 Detergent (TBST) 3 times for 15 min each. The membranes were then incubated in secondary antibody (1:2000 dilution in blocking buffer) for 1 h at room temperature, followed by three more TBST washes. After the final wash, the membrane was imaged with an Odyssey Licor imaging system (LI-COR Biotechnology, Lincoln, NE, USA).

References

1. Tyrpak, D.; Mackay, J. A., SIAL: A simple image analysis library for wet-lab scientists. *The journal of open source software* **2020,** *5*, 2689.
2. Tyrpak, D., Corrected-Total-Cell-Fluorescence. **2019**.
3. Graham, J. M., Isolation of Lysosomes from Tissues and Cells by Differential and Density Gradient Centrifugation. *Current Protocols in Cell Biology* **2000,** *7*, 3.6.1-3.6.21.
